# Work-family trajectories and health: A systematic review

**DOI:** 10.1016/j.alcr.2022.100466

**Published:** 2022-06

**Authors:** Vendula Machů, Iris Arends, Karin Veldman, Ute Bültmann

**Affiliations:** Department of Health Sciences, Community and Occupational Medicine, University Medical Center Groningen, University of Groningen, Groningen, The Netherlands

**Keywords:** Work, Family, Health, Trajectories, Life-course, Systematic review

## Abstract

**Background:**

Work and family lives interact in complex ways across individuals’ life courses. In the past decade, many studies constructed work-family trajectories, some also examined the relation with health. The aims of this systematic review were to summarise the evidence from studies constructing work-family trajectories, and to synthesise the evidence on the association between work-family trajectories and health.

**Methods:**

We searched MEDLINE, EMBASE, PsycINFO, SocINDEX and Web of Science databases. Key search terms related to work, family and trajectories. Studies that built combined work-family trajectories or examined the relationship between work and family trajectories were included. Risk of bias was assessed independently by two authors. The identified work-family trajectories were summarised and presented for men and women, age cohorts and contexts. The evidence on the association with health as antecedent or consequence was synthesised.

**Results:**

Forty-eight studies, based on 29 unique data sources, were included. Thirty-two studies (67%) were published in 2015 or later, and sequence analysis was the primary analytic technique used to construct the trajectories (n = 43, 90%). Trajectories of women were found to be more diverse and complex in comparison with men. Work-family trajectories differed by age cohorts and contexts. Twenty-three studies (48%) examined the association between work-family trajectories and health and most of these studies found significant associations. The results indicate that work-family trajectories characterised by an early transition to parenthood, single parenthood, and weak ties to employment are associated with worse health outcomes.

**Conclusions:**

Work-family trajectories differed greatly between men and women, but differences seemed to decrease in the youngest cohorts. Given the current changes in labour markets and family formation processes, it is important to investigate the work and family lives of younger cohorts. Work-family trajectories were associated with health at different life stages. Future research should examine longitudinal associations of work-family trajectories with health and focus on elucidating why and under which circumstances some trajectories are associated with better or worse health compared with other trajectories.

## Highlights

• This is the first synthesis of the evidence on work-family trajectories and health.
• Work-family trajectories differed by sex, age cohorts, and contexts.
• Trajectories of men and women become more similar in the youngest cohorts.
• Work-family trajectories were associated with health at different life stages.
• More research on the longitudinal association with health is needed.

## 1 Introduction

Work and family lives interact in complex ways across individuals’ life courses. Decisions about family formation are directly influenced by work, with labour market participation being one of the main reasons for the postponement of parenthood in both men and women (Mills, Rindfuss, McDonald, & te Velde, 2011). At the same time, having children affects decisions on parental leave or part-time work and subsequent attachment to the workforce (Kaufman, 2018, Sigurdardottir and Garðarsdóttir, 2018, Zagorsky, 2017). Work-family trajectories are often used to study how work and family lives develop during the life course. Trajectories are central to describing individual life courses and have been defined as “life course dynamics that take place over an extended period of time” (Macmillan, 2005). The recent methodological advances in building trajectories allowed for examining the timing of events, the duration that people spend in different work and family states and the ordering in which events happen when studying the relationship between work and family. Research on work-family trajectories has increased tremendously over the past decade (e.g. Aassve, Billari, & Piccarreta, 2007; Amato et al., 2008; Ice, Ang, Greenberg, & Burgard, 2020; McMunn et al., 2015; Mynarska, Matysiak, Rybińska, Tocchioni, & Vignoli, 2015).

The complex interaction between work and family events influences, and is influenced by, health. Until recently, studies primarily analysed health as a consequence of either work or family events individually (e.g. Flores & Kalwij, 2014). Overall, partnership, parenting and employment were found to be associated with better health in most studies when analysed individually (e.g. Hewitt, Baxter, & Western, 2006; Kalucza, Hammarström, & Nilsson, 2015; Willitts, Benzeval, & Stansfeld, 2004). Previous studies, however, were limited in their design to establish the timing of health, family and work events and to shed light on causal processes (McMunn, 2020). Also, the life course approach suggests that life events have differential health effects depending on the timing and the duration of these events (Kuh, Ben-Shlomo, Lynch, Hallqvist, & Power, 2003). For instance, past marital experience, including the timing of remarriage, influenced mortality and morbidity (Grundy & Tomassini, 2010), longer periods of unemployment were associated with a decrease in physical working capacity and mental health (Maier et al., 2006), and the age of children was an important factor influencing parents’ mental health (Simon & Caputo, 2019). Moreover, the two domains of work and family may have a combined impact on health. Thus, a longitudinal approach is needed to consider the interaction between work and family and its relationship with health over time. In the past decade, researchers have started to examine the relationship between combined work-family trajectories and health, for example with regards to depression (Engels et al., 2019), metabolic markers (Lacey, Kumari et al., 2016) or mortality (Sabbath, Guevara, Glymour, & Berkman, 2015).

To date, a considerable amount of research has examined combined work-family trajectories but the results have not yet been summarised. A large number of studies demonstrated that historical context, including norms around parenthood or women’s employment, shapes fertility decisions and labour market participation (e.g. Billari & Liefbroer, 2010). Similarly, many studies showed that countries’ work-family policies shape employment and family life courses (e.g. Misra, Budig, & Boeckmann, 2011). The aim of the current study was twofold. The first aim was to summarise the evidence from studies constructing work-family trajectories and to explore the differences in work-family trajectories between men and women in different cohorts and contexts. The second aim was to synthesise the evidence on the association between work-family trajectories and health either as an antecedent and/or as a consequence.

## 2 Methods

The systematic review protocol was registered with the International prospective register of systematic reviews (PROSPERO) in October 2019 under the number CRD42020152916. The systematic review is reported according to the Preferred Reporting Items for Systematic Reviews and Meta-Analyses (PRISMA) guidelines (Liberati et al., 2009).

### 2.1 Eligibility criteria

We operationalised work and family trajectories as longitudinal representations of work and family states over individual life courses. The important aspect of a trajectory is that it captures the duration of states and the transitions from one state to another (Kuh et al., 2003). Studies had to fulfil the following inclusion criteria: (1) constructing combined work-family trajectories or analysing the relationship between work and family trajectories, (2) using longitudinal data on work and family collected in a prospective study or a retrospective study, (3) defining work states as employment status (i.e. having a job or not), the number of hours worked, contract type or any other employment characteristic or a combination of characteristics, (4) defining family states as marital status, parenthood or a combination of both, (5) having no limitation on the number of possible transitions in work-family trajectories (e.g. studies that only assessed the timing of the first job or first partnership were excluded) and observing transitions between events in both possible directions (e.g. observing both getting married and getting divorced in a family trajectory), (6) building trajectories by applying a trajectory modelling technique, e.g. sequence analysis or latent class analysis (Han, Liefbroer, & Elzinga, 2017). Methodological studies that explained the application of a sequence or latent trajectory analysis and used work-family trajectories as an example were excluded when no detailed description of the results was provided (e.g. Gauthier, Widmer, Bucher, & Notredame, 2010).

### 2.2 Search

The initial systematic search was conducted on October 2nd, 2019 without a limitation on publication date or language. The following databases were searched for articles published in peer-reviewed journals: MEDLINE, EMBASE, PsycINFO, SocINDEX and Web of Science. To be eligible for inclusion, the article title and/or abstract had to contain a combination of search terms related to a) trajectory (e.g. course, pathway, pattern, class, cluster, profile), b) work (e.g. job, occupation, profession, employment, career, labour) and c) family (e.g. marriage, cohabitation, union, parent, child, life, fertility). These terms were combined with relevant database key terms (Emtree or Thesaurus). For the detailed search strategy, see Appendix 1. Further, we reviewed references of included studies to identify additional relevant articles. Upon finalising this review we conducted an updated search on April 24th, 2021.

### 2.3 Study selection

We exported search results into EndNote X9 and removed the duplicate references. References were uploaded to the Rayyan screening tool (Ouzzani, Hammady, Fedorowicz, & Elmagarmid, 2016). The study selection started with screening titles and abstracts for eligibility. In the title screening stage, all articles that did not mention terms related to work or family were excluded. During the abstract screening stage, studies were excluded that did not fulfil all inclusion criteria. In case of insufficient information to judge the inclusion criteria in the abstract, the full text was read. Full texts were obtained for detailed assessment. All references were assessed independently by two authors (VM and IA/KV/UB) in each stage of the study selection process. Discrepancies were resolved by discussion and involvement of a third author when necessary (IA/KV/UB).

### 2.4 Data extraction process

A data extraction table was developed and piloted. For each study, we extracted basic information (title, year, authors, study aim), data description (data source, country, year of data collection), sample description (size, cohort, life stage of interest, % women), methods (statistical method, unit of trajectory, operationalisation of work and family states) and results (number and names of trajectories separately for men and women). If the studies analysed the association between work-family trajectories and health, the information on the included health variables and the description of the observed association was extracted. One author (VM) extracted the data from included studies and a quality check was done for 10 studies (20.8%) for which a second author (IA) or a student assistant independently extracted data.

### 2.5 Assessment of bias

For each study, the risk of bias (RoB) was assessed with a modified version of the Quality in Prognosis Studies (QUIPS) tool (Hayden et al., 2019). The QUIPS tool was adapted to our research questions (see Appendix 2). All studies were assessed by two authors (VM and IA/KV/UB) independently; a third author (IA/KV/UB) was involved to resolve discrepancies. We assessed the RoB in six domains: selection bias, attrition bias, measurement and recall bias in assessing work and family states, measurement bias in assessing health variables (in case associations between work-family trajectories and health variables were analysed), study confounding (if applicable) and statistical analysis. The RoB in each domain was rated as low, moderate or high. The authors of six articles rated as ‘high’ in the selection bias domain due to a lack of information, were contacted and additional information was requested.

### 2.6 Methods of analysis and the synthesis of results

We summarised the identified work-family trajectories by a) sex (men, women, both); b) year of birth, referred to as an age cohort; and c) contexts (the United States (US), the United Kingdom (UK), Western Europe, other), as these aspects have shown to influence work-family trajectories (e.g. Comolli, Bernardi, & Voorpostel, 2021; Lacey et al., 2017; McDonough, Worts, Booker, McMunn, & Sacker, 2015). The division of contexts was based on the unequal geographical distribution of the identified studies with most studies (56.3%) analysing samples from the UK and the US. We focused on the differences in timing, ordering and duration of the important work and family events in the identified trajectories. Results from studies examining the association between work-family trajectories and health were synthesised based on whether health was assessed as antecedent or consequence of the work-family trajectories. We operationalised health as any assessment of physical or mental well-being or the presence/absence of physical or mental disease (e.g. cardiovascular disease, depressive symptoms, self-rated health).

## 3 Results

**Fig. 1:**
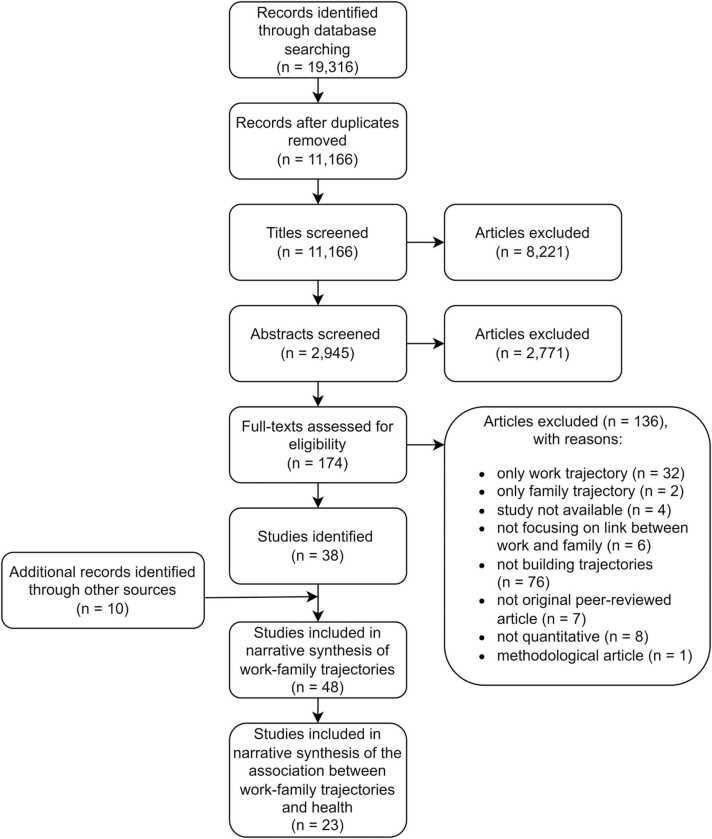
Flow diagram.

The database search resulted in the identification of 11,166 unique references. In the title screening, 8221 references were excluded, often because key search terms had a different connotation, e.g. labour in the meaning of childbirth. During the abstract screening, another 2771 articles were excluded, mostly because no trajectories were built or the focus was not on the association between work and family trajectories. Another 136 articles were excluded in the full-text screening, mainly because no trajectories (n = 76) or only work trajectories (n = 32) were built. Ten additional articles were identified through screening the references of included studies. The full selection process is shown in the flow diagram in Fig. 1.

A total of 48 studies met all the inclusion criteria. Among these 48 studies, six pairs of studies presented identical typologies of work-family trajectories but addressed different research questions regarding antecedents and consequences of the trajectories: Amato et al. (2008) and Amato and Kane (2011); Huang, El-Khouri, Johansson, Lindroth, and Sverke (2007) and Johansson, Huang, and Lindfors (2007); McMunn et al. (2016) and Lacey, Sacker et al. (2016); Oesterle, Hawkins, Hill, and Bailey (2010) and Oesterle, Hawkins, and Hill (2011); Salmela-Aro et al., 2011, Salmela-Aro et al., 2014; Lacey, Kumari et al. (2016) and Stafford et al. (2019). Further, Mayeda et al. (2020) and Sabbath et al. (2015) presented the same work-family typology, but in the analysis of the association with health outcomes, Mayeda et al. (2020) used a simplified set of five, instead of seven trajectories.

### 3.1 Characteristics of included studies

Of the 48 studies that built work-family trajectories, the majority (n = 32, 66.7%) was published in 2015 or later. For the main characteristics of the included studies see Table 1; a detailed description of each included study is provided in Appendix 3.

**Table 1:** Sample and analytical details of 48 studies included in the review.

	Country	Year of birth	N	Trajectories in age range	Women (%)	Statistical method
Aassve et al. (2007)	UK	1960–1969	578	13–30	100	SA
Aeby et al. (2019)	Switzerland	1970–1975	382	16–41	53.0	SA
Aisenbrey and Fasang (2017)	US, Germany	1957–1964 1956–1965	8630	22–44	not reported	SA
Amato et al. (2008)	US	1976–1979	2290	18–23	100	LCA
Amato and Kane (2011)	US	1976–1979	2290	18–23	100	LCA
Arpino et al. (2018)	Snot reported	before 1949	12,034	15–59	51.7	SA
Barnett (2013)	US	1931–1941	1300	51–75	79.0	LCA
Bennett and Waterhouse (2018)	South Africa	1991–1993	429	15–24	100	SA
Carmichael and Ercolani (2016)	UK	1906–1980	4339	16–85 at the beginning, followed for 15–20 years	56.0	SA
Comolli et al. (2021)	Switzerland	1933–1966	1885	20–50	53.3	SA
Davia and Legazpe (2014)	Spain	1956–1970	1946	16–35	100	SA
Engels et al. (2019)	Germany	1925–1955	3019	20–50	50.9	SA
Huang et al. (2007)	Sweden	1955	549	16–43	100	SA
Ice et al. (2020)	14 SHARE countries^1^	1930–1957	11,908	12–50	100	SA
Jin et al. (2020)	US	1930–1983	569	20–35	49.0	SA
Johansson et al. (2007)	Sweden	1955	549	16–43	100	SA
Koelet et al. (2015)	Belgium	1976	1598	14–29	49.6	SA
Lacey, Kumari et al. (2016)	UK	1946	2503	16–51	50.0	SA
Lacey, Sacker et al. (2016)	UK	1958	7228	16–42	51.1	SA
Lacey, Stafford et al. (2016)	UK	1946	2000	16–60	not reported	SA
Lacey et al. (2017)	UK	1946 1958 1970	20,760	16–42	1946: 50.6 1958: 51.3 1970: 53.3	SA
Madero-Cabib and Fasang (2016)	Switzerland, Germany	1920–1950	1709	20–59	55.1 48.8	SA
Madero-Cabib et al. (2016)	Switzerland	before 1951	674	20–57	41.1	SA
Mayeda et al. (2020)	US	1935–1956	6189	16–50	100	SA
McDonough et al. (2015)	US, UK	1957–1964 (US) 1958 (UK)	8455	25–39	100	SA
McKetta et al. (2018)	US	1927–1978	6039	18–50	100	SA
McMunn et al. (2015)	UK	1946 1958 1970	20,786	16–42	1946: 50.6 1958: 51.3 1970: 53.3	SA
McMunn et al. (2016)	UK	1958	7228	16–42	51.1	SA
Mooyaart et al. (2019)	US	1980–1984	4688	17–27	52.9	SA
Müller et al. (2012)	Switzerland	not reported	86	16–34	49.0	SA
Mynarska et al. (2015)	Italy, Poland	1965–1974	920	15–37	100	SA
Oesterle et al. (2010)	US	1975	728	18–30	50.3	LCA
Oesterle et al. (2011)	US	1975	728	18–30	50.3	LCA
Pailhé (2013)	France	1954–1968	941 couples	18–49	NA	SA
Piccarreta and Billari (2007)	UK	1960–1968	578	13–30	100	SA
Pollock (2007)	UK	not reported	5124	calendar years 1991–2000	not reported	SA
Sabbath et al. (2015)	US	1936–1956	7536	16–50	100	SA
Salmela-Aro et al. (2011)	Finland	1966–1973	182	18–43	78.0	SA
Salmela-Aro et al. (2014)	Finland	1966–1973	182	18–43	78.0	SA
Scherger et al. (2016)	UK	1916–27 1928–37 1938–47 1948–57	6334	15–50	52.7	SA
Sirniö et al. (2017)	Finland	1972–1975	23,915	16–37	48.5	SA
Stafford et al. (2019)	UK	1946	2513	16–51	50.0	SA
Tocchioni (2018)	Italy	1907–1969	3414	16–50	49.4	SA
Van Hedel et al. (2016)	US, 13 SHARE countries^2^	1935–1956	18,250	16–50	100	SA
Vidal et al. (2020)	Germany	1930–1949 1958–1981	1246	18–35	100	SA
Worts et al. (2013)	US	1942–1945 1946–1949 1950–1953 1957– 1960 1961–1964	7150	25–49	100	SA
Xue et al. (2020)	UK	before 1956	3889	14–26	100	SA
Zimmermann (2021)	Germany	1920–1957	2542	18–60	100	SA

UK, United Kingdom; US, United States LCA, latent class analysis; SA, sequence analysis

^1^SHARE countries: Austria, Belgium, Czech Republic, Denmark, France, Germany, Greece, Ireland, Italy, Netherlands, Poland, Spain, Sweden, and Switzerland

^2^SHARE countries: Austria, Belgium, Czech Republic, Denmark, France, Germany, Greece, Italy, Netherlands, Poland, Spain, Sweden, and Switzerland

#### 3.1.1 Study design

The 48 studies used data from 29 unique data sources. Twenty-five studies (52.1%) built work-family trajectories by analysing prospectively collected data (e.g. British Household Panel Survey, National Longitudinal Survey of Youth or National Longitudinal Survey of Women) or register-based data. Twenty-one studies (43.8%) analysed data collected retrospectively at one time point with a life event history calendar, e.g. the third wave of the SHARE (The Survey of Health, Ageing and Retirement in Europe) study, Italian Multipurpose Household Survey on Family and Social Subjects or Helsinki Longitudinal Student Study. Two studies (4.2%) used a combination of data collected retrospectively at one time point and data collected in multiple waves (Aisenbrey and Fasang, 2017, Van Hedel et al., 2016).

#### 3.1.2 Sample characteristics

In 19 studies (39.6%), work-family trajectories were constructed only for women. In 28 studies (58.3%), the trajectories were built for both men and women, but in four studies the results were not reported separately for men and women. One study built combined work-family trajectories of couples (Pailhé, 2013).

Nineteen studies (39.6%) built trajectories in samples born across multiple decades of the 20th century. Five studies (10.4%) compared trajectories of people born in different decades. Respondents of seven studies (14.6%) were born before 1951, respondents of four studies (8.3%) were born in the 1950s, respondents of two studies (4.2%) were born in the 1960s, and respondents of seven studies (14.6%) were born in the 1970s. Finally, two studies (4.2%) built trajectories for the generation of millennials born after 1981 (Bennett and Waterhouse, 2018, Mooyaart et al., 2019). Two studies (4.2%) did not report the birth year of the sample.

The majority of studies analysed samples from either the UK (n = 14, 29.2%) or the US (n = 14, 29.2%), and one of these studies compared the trajectories of US and UK women (McDonough et al., 2015). Out of the 14 studies building trajectories for a US sample, two studies made a comparison with other samples: one study compared the trajectories of a US sample with a German sample (Aisenbrey & Fasang, 2017) and the other study compared a US sample to the pooled sample of countries included in the SHARE survey (Van Hedel et al., 2016). The pooled sample of SHARE countries was analysed in two other studies (Arpino et al., 2018, Ice et al., 2020). Eighteen studies (37.5%) constructed trajectories for European countries: Belgium, Finland, France, Germany, Italy, Poland, Spain, Sweden and Switzerland. One study analysed a sample of young adults from South Africa (Bennett & Waterhouse, 2018).

#### 3.1.3 Analytical approach to building trajectories

The vast majority of included studies (n = 43, 89.6%) used sequence analysis (SA) to build work-family trajectories. In most of these studies (n = 34, 70.8%), the authors constructed sequences of individual work and family life courses and subsequently applied a clustering method to create a typology of the most common work-family trajectories. In studies that analysed trajectories in both men and women, clustering was either done separately for men and women (e.g. Oesterle et al., 2010) or together for both sexes (e.g. Scherger, Nazroo, & May, 2016). The remaining nine studies that applied sequence analysis, used a pre-defined set of theoretically informed work-family trajectories (e.g. McMunn et al., 2015; Stafford et al., 2019). Five studies (10.4%) used latent class analysis (LCA) to identify distinct work-family trajectories.

The included studies used data with a different level of detail, varying from monthly to biennial periods. Most studies started building trajectories from adolescence or young adulthood (most often around the ages 16–18) and covered different lengths of individual trajectories up till age 75. Nine studies analysed trajectories in young adulthood, that is till age 30 (e.g. Aassve et al., 2007), and one study built trajectories in late adulthood starting at 51–61 years (Barnett, 2013) (for details on all included studies see Table 1). Different variables were used to define work and family states. In some studies, the family states were binary, e.g. married and not married, parent and non-parent (e.g. Engels et al., 2019). In other studies, the ordering of the children, the age of the children or the ordering of the unions were taken into account (e.g. Lacey et al., 2017; Müller, Sapin, Jacques-Antoine, Orita, and Widmer, 2012). The possible work states also varied from binary (e.g. employed, not employed) to more detailed descriptions (e.g. the number of work hours, contract type, reasons for not being employed, job prestige). In seven studies, additional variables, next to work and family states, were included in the trajectories, namely informal caregiving, pension investment and living situation (e.g. Barnett, 2013; Sirniö, Kauppinen, & Martikainen, 2017).

#### 3.1.4 Health as antecedent or consequence of work-family trajectories

Twenty-three out of the 48 studies (47.9%) examined the relationship between work-family trajectories and health. Seven of these 23 studies (30.4%) analysed physical health operationalised as metabolic or stress markers, inflammation, obesity, or heart disease. Eight out of the 23 studies (34.8%) assessed general health, including self-rated general health and mortality. Thirteen out of the 23 studies (56.5%) analysed mental health operationalised as depression, substance use, well-being, cognitive impairment, memory decline and psychiatric disorders. Several studies examined multiple health variables.

Twenty-one out of the 23 studies (91.3%) analysed health as a consequence of work-family trajectories (e.g. Bennett & Waterhouse, 2018; Huang et al., 2007), and five studies (21.7%) analysed health as an antecedent of work-family trajectories (e.g. Arpino et al., 2018; Salmela-Aro et al., 2014). Four studies not only analysed the association with health at one point, but examined the association of work-family trajectories with longitudinal change in health (e.g. Mayeda et al., 2020; Van Hedel et al., 2016). Some studies examined health at multiple time points, e.g. assessed health both as an antecedent and consequence of work-family trajectories (e.g. Amato & Kane, 2011).

A study by Amato et al. (2008) was not included in the summary of the relationship between health and work-family trajectories, because health was analysed as a part of the factor *personal and social resources* and not as a separate precursor of work-family trajectories. A later study by Amato and Kane (2011) explored the association of the same set of trajectories with depression; this study was included in the synthesis.

#### 3.1.5 Risk of bias

A summary of the Risk of Bias (RoB) assessment across the studies is presented in Table 2 (RoB assessment per study available on request). We assessed 20 studies (41.7%) with a low/moderate risk of bias in all domains. The risk of selection bias was most often rated as moderate due to low response rates, not providing enough details about the sample selection and lack of reporting on the baseline characteristics. Two studies (4.2%) were rated as high risk of selection bias, due to not providing information on recruitment, attrition and baseline characteristics (additional information was requested from the authors, but no response was received). Regarding studies with moderate (n = 21, 43.8%) or high (n = 24, 50.0%) risk of attrition bias, authors did not report how the final analytical sample was selected, how many respondents were excluded from the analysis and how this could have affected the results. The risk of measurement bias was mostly low. In studies that used data with a long recall period, the risk of recall bias was rated as moderate (e.g. Salmela-Aro et al., 2011). An aspect that was not often covered was how authors handled missing data on work, family, health and potential confounders. Two studies did not clearly describe or adjust for possible confounders in the relationship between work-family trajectories and health. Also, multiple studies did not account for prior health when analysing the association between work-family trajectories and subsequent health. Studies that did account for prior health usually adjusted for prior health in the analysis (e.g. (Mooyaart et al., 2019) or excluded participants with prior health problems from the analysis (e.g. Engels et al., 2019). The risk of statistical and reporting bias was rated as low in all included studies. The details of the description differed, but all studies reported on all important aspects of the analysis.

**Table 2:** Risk of bias assessment across six domains in 48 included studies.

	Selection bias	Attrition bias	Measurement and recall bias: work and family	Measurement bias: health	Confounding	Statistical analysis and reporting
Aassve et al. (2007)						
Aeby et al. (2019)						
Aisenbrey and Fasang (2017)						
Amato et al. (2008)						
Amato and Kane (2011)						
Arpino et al. (2018)						
Barnett (2013)						
Bennett and Waterhouse (2018)						
Carmichael and Ercolani (2016)						
Comolli et al. (2021)						
Davia and Legazpe (2014)						
Engels et al. (2019)						
Huang et al. (2007)						
Ice et al. (2020)						
Jin et al. (2020)						
Johansson et al. (2007)						
Koelet et al. (2015)						
Lacey, Kumari et al. (2016)						
Lacey, Sacker et al. (2016)						
Lacey, Stafford et al. (2016)						
Lacey et al. (2017)						
Madero-Cabib and Fasang (2016)						
Madero-Cabib et al. (2016)						
Mayeda et al. (2020)						
McDonough et al. (2015)						
McKetta et al. (2018)						
McMunn et al. (2015)						
McMunn et al. (2016)						
Mooyaart et al. (2019)						
Müller et al. (2012)						
Mynarska et al. (2015)						
Oesterle et al. (2010)						
Oesterle et al. (2011)						
Pailhé (2013)						
Piccarreta and Billari (2007)						
Pollock (2007)						
Sabbath et al. (2015)						
Salmela-Aro et al. (2011)						
Salmela-Aro et al. (2014)						
Scherger et al. (2016)						
Sirniö et al. (2017)						
Stafford et al. (2019)						
Tocchioni (2018)						
Van Hedel et al. (2016)						
Vidal et al. (2020)						
Worts et al. (2013)						
Xue et al. (2020)						
Zimmermann (2021)						

Low risk of bias  Moderate risk of bias  High risk of bias.

### 3.2 Synthesis of results

The identified trajectories in each of the 48 studies are presented in Appendix 4. The studies identified between three and twelve trajectories. In most studies (n = 46, 95.8%), trajectories were presented by assigning a short title capturing the most significant characteristics of the trajectory, e.g. *full-time worker, early union formation without children* (Koelet, de Valk, Glorieux, Laurijssen, and Willaert, 2015).

#### 3.2.1 Work-family trajectories

##### 3.2.1.1 Men and women

Twenty-four studies (50.0%) analysed differences in work-family trajectories between men and women. Overall, the between-person diversity was higher in women, which means that their trajectories were less similar in comparison with men. The lower diversity of men’s trajectories was also reflected by a lower number of identified trajectories. For example, Arpino et al. (2018) identified six trajectories in men and 12 trajectories in women. In studies that built trajectories of men and women together, men often belonged to a smaller number of trajectories wr market. An exception was the. Lacey, Sacker et al., 2016; Sirniö et al., 2017). For example, Stafford et al. (2019) identified eight trajectories in men and women, but 49.9% of men, as opposed to 30.7% of women, belonged to the one most common trajectory. Furthermore, women’s work-family trajectories were found to be more complex with more transitions between different work and family states, i.e. within-person diversity was higher among women compared with men (e.g. Engels et al., 2019; McMunn et al., 2015).

In men, the most common work-family trajectory across the studies was represented by uninterrupted employment with varying timing of family formation (e.g. Comolli et al., 2021). For example, the trajectories identified by Scherger et al. (2016) show that men were more likely to have children later without interrupting their career (34.5% of men vs. 9.5% of women) or take only a short break from work after starting a family early (39.9% of men vs. 28.4% of women). In a study by Koelet et al. (2015), 32.0% of men, compared with 51.0% of women, followed trajectories characterised by early family formation. Continuous full-time employment was found to be the main characteristic in men’s trajectories across studies, e.g. 92.0% of men compared with 74.0% of women (Koelet et al., 2015). In a study by Arpino et al. (2018), only 2.1% of men versus 40.8% of women were in trajectories characterised by being inactive at the labour market. An exception was the study by Mooyaart et al. (2019), in which the most common trajectory in young men (29.2% of men) was characterised by unstable employment and the trajectories describing stable employment were as common in men as in women (32.3% and 33.6%, respectively). Similarly, the study by Jin et al. (2020) did not find many differences between the trajectories of men and women.

Women’s trajectories were more often characterised by an earlier family formation and career breaks compared with men (e.g. Aeby, Gauthier, & Widmer, 2019; Koelet et al., 2015; Madero-Cabib & Fasang, 2016). For example, Engels et al. (2019) found that almost half of the women had trajectories characterised by part-time work, whereas there was no such trajectory identified in men. Similarly, Lacey, Kumari et al. (2016) found that 29.9% of women and 0.7% of men belonged to the trajectory *part-time work, early family*. In a study that included occupational prestige in the analysis of work-family trajectories, women were more likely than men to experience single parenthood and unstable low-prestige work at the same time (20.0% of women and 12.0% of men in the trajectory *single, children, disrupted low prestige*) (Aisenbrey & Fasang, 2017). Tocchioni (2018) investigated trajectories of childless people and found that the most common trajectory in both men and women was *employed single* (35.0% of men vs. 32.5% of women). In comparison with men, women were more often in a *disadvantaged* trajectory (8.9% of men vs. 17.3% of women) and the author also identified a women-only *stay-at-home wives* trajectory (16.3% of women).

##### 3.2.1.2 Cohorts

Differences between cohorts were especially noticeable in women, as labour market participation increased and breaks from work became shorter in younger cohorts (e.g. Scherger et al., 2016). Vidal, Lersch, Jacob, and Hank (2020) found that the most common trajectory of women born between 1930 and 1949 was *stay-at-home mothers* (38.5% of women), whereas the most common trajectory of their daughters, that is women born in 1958–1981, was *late family formation* characterised by long spells of employment combined with later parenthood (35.4% of women). Similarly, McKetta, Prins, Platt, Bates, and Keyes (2018) showed that the most common trajectory was *non-working, married, earlier mothers* in women born between 1920 and 1939, *non-working, married, late mothers* in women born between 1940 and 1959 and *working, divorced mothers* in women born between 1960 and 1979. In a sample of childless people, labour market participation also increased across cohorts in women but remained stable in men (Tocchioni, 2018). Later born respondents were more evenly spread across the trajectories, i.e. the diversity in work-family trajectories increased with each consecutive cohort (McMunn et al., 2015, Worts et al., 2013). Also, when comparing people born in 1946, 1958 and 1970, the differences were less pronounced between the trajectories of men and women in the youngest cohort (McMunn et al., 2015). Trajectories characterised by full-time employment were as common in men as in women in young adults born in 1980–1984 (Mooyaart et al., 2019).

The complexity of partnership trajectories increased, as people from younger cohorts more often cohabited, divorced and started new unions (McMunn et al., 2015, Worts et al., 2013). Parental trajectories became less complex, possibly due to having fewer or no children. Jin et al. (2020) found that the inter-generational differences in work-family trajectories are mostly due to the timing of the childbirth, i.e. people born earlier more often followed the trajectories *family first* and *have-it-alls* when compared with younger people. Trajectories characterised by early parenthood in both men and women became less prevalent in younger generations when comparing people born in 1946, 1958 and 1970 (Lacey et al., 2017). While women’s employment trajectories became less complex, as they were more often characterised by consistent work participation (Lacey et al., 2017, Scherger et al., 2016, Worts et al., 2013), the complexity of men’s employment trajectories increased due to higher occurrences of unemployment (McMunn et al., 2015).

##### 3.2.1.3 Contexts

Five studies (10.4%) compared work-family trajectories in different geographical contexts. Three studies compared work-family trajectories of participants from the US with those from European countries, specifically Germany (Aisenbrey & Fasang, 2017), the UK (McDonough et al., 2015) and a pooled sample of 13 European countries (Van Hedel et al., 2016). In the German sample, larger differences in work-family trajectories were identified between men and women compared with the US sample (Aisenbrey & Fasang, 2017). Van Hedel et al. (2016) found that US women were more likely to experience the trajectory *working single mothers* than European women (11.3% of US women vs 5.5% of European women). The main difference between the US and the UK samples of women was the frequency of continuous full-time and part-time employment. US women were more often in the trajectory *married mother full-timer* compared with the UK women (40.7% vs 28.6%, respectively) and less often in the trajectory *married mother part-timer* (11.9% vs 25.0%, respectively) (McDonough et al., 2015). A comparison between Swiss and German samples showed that the differences in identified work-family trajectories between the two countries were marginal (Madero-Cabib & Fasang, 2016). Lastly, one study compared work-family trajectories of Italian and Polish childless women (Mynarska et al., 2015). In the Italian sample, the trajectory *low-educated single working women* was dominant (42.3%), while Polish women were evenly distributed among the six identified trajectories.

#### 3.2.2 Work-family trajectories and health

Twenty-three studies examined the association between work-family trajectories and health at different life stages. We have synthesised the evidence on the association between work-family trajectories and seven health variables examined across multiple studies, i.e. depression, cognitive impairment, mental distress, metabolic markers, obesity, self-rated general health and mortality. For the complete overview of the main results of the included studies, see Table 3.

**Table 3:** Association between work-family trajectories and health variables in 23 included studies.

	Relationship of health with work-family trajectories	Results
**Mental health**		
Depression	Antecedent	Salmela-Aro et al. (2014): *Slow starters* and *singles with slow careers* reported more depressive symptoms compared with the other trajectories
	Consequence	Engels et al. (2019): In men, there was no association between work-family trajectories and depression; in women, the trajectory *re-entry in full-time work, children* was associated with more depressive symptoms and intake of antidepressant medication compared with the trajectory *re-entry in full-time work, children* (participants with early depression were excluded from the analysis) McDonough et al. (2015): The trajectories *divorcing back-to-work mother*, *single at-home mothers* and *married at-home mother* were associated with a greater risk of depression than the trajectory *married mother full-timer,* adjusted for prior work-related health limitation Salmela-Aro et al. (2014): The trajectories *slow starters* and *singles with slow careers* reported more depressive symptoms compared with *non-postponed pathway,* adjusted for the initial level of depression Xue et al. (2020): The trajectories *early married parenthood early domestic labor* and *mixed family, some part-time* were associated with higher levels of depressive symptoms compared with *later marriage early full-time* group, but the association was entirely explained by the lower levels of income and wealth, not adjusted for prior depression
	Association over time	Amato and Kane (2011): Lower levels of depression before the start of the trajectory were associated with the trajectory *college to job no family-formation* compared with all other trajectories (except for the trajectories *inactive* and *high school to full-time job*); higher levels of depression before the trajectory were associated with the trajectory *single mothers* compared with trajectories *inactive* and *high school to full-time job*; all trajectories were associated with a decline in depression levels over time; the trajectory *college to job no family-formation* was associated with the lowest levels of depression after the trajectory compared with all other trajectories
Well-being	Consequence	Johansson et al. (2007); Lacey, Stafford et al. (2016): No statistically significant association between trajectories and well-being was identified
Cognitive impairment	Consequence	Ice et al. (2020): The trajectory *part-time working mothers* was associated with the best cognitive functioning and the trajectory *unpaid caregiver mothers* was associated with the lowest cognitive functioning compared with the trajectory *full-time working mothers*, not adjusted for prior cognitive functioning Mayeda et al. (2020): The trajectories *nonworking single mothers* and *nonworking married mothers* were associated with greater memory decline after age 60 compared with the trajectory *working married mothers*, not adjusted for prior memory function
Substance use disorders	Association over time	Oesterle et al. (2011): In women, there were no differences between the trajectories in alcohol abuse or dependence. In men, *unmarried men with limited postsecondary education* had higher rates of alcohol abuse or dependence than *married men* at all ages except age 30. *Unmarried early mothers* and *unmarried men with limited postsecondary education* had higher rates of nicotine dependence than other trajectories at all ages. The trajectory *unmarried men with limited postsecondary education* was associated with the highest rates of marijuana abuse and dependence compared with both other trajectories at each time point. The differences between trajectories were constant across young adulthood and were already observed before the beginning of the trajectory
Mental distress	Consequence	Johansson et al. (2007): The trajectory *working mothers* was associated with more mental distress compared with the trajectories *full timers, delayed family builders, early mothers full-time* and *Scandinavian family builders*, not adjusted for prior mental distress Lacey, Stafford et al. (2016): No statistically significant association between work-family life course types and mental distress was identified
	Association over time	Carmichael and Ercolani (2016): The trajectory *caring intensive* was associated with mental distress at the beginning of the trajectory compared with the trajectory *full-time careers*, and the differences between the trajectories *full-time careers* and *caring intensive* widened over time as those in the *caring intensive* trajectory experienced an increase in mental distress
Psychiatric disorders	Consequence	Müller et al. (2012): In people with severe mental health disorders undergoing treatment, the trajectory *standard life course with few institutionalization periods* was associated with more psychiatric symptoms and distress compared with an *institutionalized life* trajectory
**Physical health**		
Metabolic markers*	Consequence	Johansson et al. (2007): No significant association between trajectories and markers was identified Lacey, Kumari et al. (2016): In men, the trajectory *work, later family* was associated with smaller waist circumferences, lower triglycerides and lower blood pressure compared with the trajectory *work, early family*; the trajectory *work, marriage, non-parent* was associated with increased high-density lipoprotein cholesterol in men and with lower waist circumferences in women compared with the trajectory *work, early family*, not adjusted for prior levels of metabolic markers Lacey, Sacker et al. (2016): The trajectory *teen parent* was associated with higher C-reactive protein and fibrinogen levels when compared with *work, later family* trajectory; the trajectory *later family, work break* was associated with higher cortisol values than trajectory *work, later family* but no associations were found for other trajectories and cortisol, not adjusted for prior levels of metabolic markers McMunn et al. (2016): Trajectories characterised by earlier transition into parenthood were associated with significantly higher metabolic risk, regardless of work or marital stability, not adjusted for prior levels of metabolic markers
Obesity (BMI)	Consequence	Mooyaart et al. (2019): In women, trajectories characterised by college education, early home leaving and postponement of family formation were associated with a lower probability of becoming obese when compared with the trajectory *unstable employment-parental home*; in men, trajectories characterised by early marriage were associated with a higher probability of becoming obese when compared with the trajectory *unstable employment-parental home*, adjusted for prior obesity Van Hedel et al. (2016): The trajectory *nonworking married mothers* was associated with higher odds of being obese when compared with trajectory *working married mothers*, not adjusted for prior obesity
	Association over time	Lacey et al. (2017): Trajectories characterised by earlier transitions to parenthood and weaker ties to paid work were associated with larger increases in BMI over the adult life course when compared with people following other trajectories
Cardiovascular disease	Consequence	Van Hedel et al. (2016): The trajectory *working single childless women* was associated with lower odds of having high blood pressure compared with the trajectory *working married mothers*; the trajectory *working single mothers* was associated with higher odds of heart disease and stroke compared with the trajectory *working married mothers*; the trajectory *married mothers who returned to work after some non-employment* was associated with higher odds of stroke compared with the trajectory *working married mothers,* not adjusted for prior cardiovascular health
**General health**		
Self-rated health	Antecedent	Arpino et al. (2018): In women, poor health in childhood was associated with the trajectories *inactive no union; no union, children* and *employed married, no children* compared with the trajectory *married, two children*; in men, poor health in childhood was associated with the trajectories *no union, children* and *married, no children* compared with trajectory *married, two children*
	Consequence	Bennett and Waterhouse (2018): The trajectories *non-activity commonly followed by motherhood; motherhood combined with schooling;* and *motherhood after schooling* were associated with poorer self-rated health compared with the trajectories *pathway from school, motherhood then work* and *schooling to non-activity*, adjusted for prior health Huang et al. (2007): The trajectory *Scandinavian family builders* was associated with higher levels of health compared with the trajectory *working mothers*, not adjusted for prior health McDonough et al. (2015): The trajectories *married at-home mother* and *single at-home mother* were associated with worse self-rated health compared with the trajectory *married mother full-timers*, adjusted for prior work-related health
	Association over time	Amato and Kane (2011): Higher levels of general health before the trajectory were associated with the trajectory *college to job no family-formation* compared with all other trajectories (except for the trajectory *inactive*); lower levels of general health before the trajectory were associated with the trajectory *single mothers* compared with *high school to full-time job* and *inactive*; all trajectories were associated with an increase in general health over time health; the trajectory *college to job no family-formation* was associated with better later health compared with the trajectories *high school to job with no family formation, cohabiting without children, married mothers, single mothers* and *cohabiting mothers* Carmichael and Ercolani (2016): The trajectories *caring intensive* and *decaying careers* were associated with poorer health at the beginning of the trajectory compared with the trajectory *full-time careers*, and the differences between the trajectories *full-time careers* and *part-time careers* narrowed over time whereas the differences between *full-time careers* and *caring intensive,* and *full-time careers* and *decaying careers* widened over time
Mortality	Consequence	McKetta et al. (2018): The trajectory *non-working, married, later-mothers* had the lowest mortality rate; the trajectories *working, never-married non-mothers* and *working and non-working, never-married mothers* were associated with the highest mortality rate compared with the trajectory *non-working, married, later-mothers,* not adjusted for prior health Sabbath et al. (2015): The trajectory *married mother who went back to work earlier* had the lowest mortality rate; the trajectories *nonworking single mother, working single mother* and *nonworking married mother* were associated with the highest mortality rate compared with the trajectory *married mother who went back to work earlier*, associations partially explained by health behaviour

*List of markers: Johansson et al. (2007): systolic and diastolic blood pressure, total cholesterol and high-density lipoproteins, glycosylated haemoglobin, expiratory flow, waist/hip ratio

Lacey, Kumari et al. (2016): waist circumference, blood pressure, high-density lipoprotein cholesterol, triglycerides, glycated haemoglobin

Lacey, Sacker et al. (2016): inflammation (C-reactive protein, fibrinogen and von Willebrand factor), cortisol

McMunn et al. (2016): waist circumference, systolic and diastolic blood pressure, high-density lipoprotein cholesterol, triglycerides, glycated haemoglobin

##### 3.2.2.1 Depressive symptoms

The evidence on the association between work-family trajectories and depressive symptoms suggests that both the work and family components are associated with the level of depressive symptoms. Women who were single mothers, both employed and unemployed, reported higher levels of depressive symptoms across studies (Amato & Kane, 2011; McDonough et al., 2015). Also, trajectories characterised by motherhood combined with no employment were associated with more depressive symptoms (McDonough et al., 2015, Xue et al., 2020). Mothers who worked full-time reported higher levels of depression, compared to mothers who worked part-time (Engels et al., 2019). When looking at work-family trajectories in young adulthood in a selective sample of Finnish university students, higher levels of depression were observed in those who prolonged their university studies, transitioned later to working life and either remained single or formed family later, even after controlling for the initial level of depression (Salmela-Aro et al., 2014).

##### 3.2.2.2 Cognitive impairment

Two studies examined the association between work-family trajectories and cognitive impairment measured as trajectories of cognitive decline between ages 55 and 80 years in a US sample (Mayeda et al., 2020), and cognitive performance at one point between the age of 50 and 77 years in a European sample (Ice et al., 2020). Continuous employment, regardless of partnership or parenting experiences, was associated with the highest levels of cognitive functioning (Ice et al., 2020, Mayeda et al., 2020). Timing of employment across the life course did not appear to matter, as rates of memory decline were similar for married working mothers who consistently worked and those who took a break from work after childbirth (Mayeda et al., 2020). Further, Ice et al. (2020) distinguished between part-time and full-time employment in the work-family trajectories and concluded that women who mainly worked part-time had better cognitive health than women who worked full-time, even after adjusting for childhood socioeconomic disadvantage and educational status.

##### 3.2.2.3 Mental distress

The evidence on the association between work-family trajectories and mental distress is inconclusive. Lacey, Stafford, Sacker, and McMunn (2016) did not observe any significant association between work-family trajectories and mental distress. Carmichael and Ercolani (2016) did not find baseline differences in mental distress for different work-family trajectories among British women. Differences in mental distress that were observed during and at the end of the trajectories, were mostly due to the level of intensity of caregiving. In contrast, Johansson et al. (2007) found that Swedish working mothers with limited education who entered the labour market early and gave birth late, reported slightly higher levels of mental distress compared with other work-family trajectories. The authors explain the modest difference by the fairly healthy sample and the context in which these women experienced important life transitions, specifically the improvement of the Swedish welfare system between the 1970s and 1990s that supported women’s labour market participation and gave them better control of their work and family lives.

##### 3.2.2.4 Metabolic markers

Johansson et al. (2007) did not find any association between work-family trajectories and metabolic markers, possibly due to a quite healthy sample. Later parenthood combined with continuous full-time employment and marriage was associated with a more favourable metabolic risk profile in men, but not in women (Lacey, Kumari et al., 2016). Work-family trajectories characterised by weaker ties to paid work and an early transition into parenthood, including teenage parenthood, were associated with later chronic inflammation (Lacey, Sacker et al., 2016) and increased metabolic risk (McMunn et al., 2016). These associations were largely explained by a less healthy lifestyle among participants with weaker ties to work and earlier transitions to parenthood in the study by Lacey, Sacker et al. (2016), but only partially attenuated after adjusting for education, early health, health behaviours, BMI and social class in the study by McMunn et al. (2016).

##### 3.2.2.5 Obesity

Parenthood and weak ties to employment were associated with a higher risk of obesity. Lacey et al. (2017) found an association between early parenthood combined with weak ties to employment and obesity consistently across three British cohorts in both men and women, even after adjusting for birth weight, child BMI, prior health, educational attainment and socioeconomic position. The analysis of the US and European samples showed a higher risk of obesity in nonworking married mothers compared with working married mothers (Van Hedel et al., 2016). However, in the current generation of the US young women, combining working and parenting was associated with the highest risk of obesity, whereas women who stayed in education, postponed partnership and parenthood had the lowest risk of being obese (Mooyaart et al., 2019). In young men, higher levels of education lowered the risk of becoming obese, however not when combined with early marriage. Men who married early and did not have children had the highest risk of obesity in young adulthood. The different findings in the UK population (Lacey et al., 2017) and the current generation of young adults in the US (Mooyaart et al., 2019) might be a consequence of the major differences in the characteristics of the samples regarding the socio-political and historical contexts and the analysed life stage, i.e. focusing on young adulthood only vs. on the entire adulthood.

##### 3.2.2.6 Self-rated general health

Evidence on the association between early health problems and subsequent work-family trajectories is inconclusive. Poor health in childhood was associated with no family formation and long time spent in employment (Arpino et al., 2018). Poor health in adolescence was associated with single motherhood in young adulthood, mostly combined with employment at age 23 (Amato & Kane, 2011). Several studies examined the association between work-family trajectories and subsequent general health. In the US and UK, single unemployed women were most likely to report worse health (McDonough et al., 2015). Similarly, people whose trajectories were characterised by full-time employment and no caregiving responsibilities reported better health than those whose trajectories were characterised by intensive caregiving out of employment (Carmichael & Ercolani, 2016). Swedish women with different work-family trajectories did not differ in general health with one exception, i.e. women with trajectories of full-time employment followed by part-time employment after childbirth and a subsequent return to full-time employment reported better health in comparison with women who mostly worked full-time during the trajectory and only later in the trajectory experienced various work or family transitions (Huang et al., 2007).

Two studies examined the association between general health and work-family trajectories in early young adulthood (Bennett & Waterhouse, 2018, Amato & Kane, 2011). In both studies, a longer education period and a later transition to employment were associated with the highest level of general health. South African women who followed trajectories characterised by early parenthood reported poorer general health, but only when they were unemployed (Bennett & Waterhouse, 2018). Mothers who were employed reported better health than unemployed mothers, even after adjusting for baseline socio-economic and demographic characteristics and health (Bennett & Waterhouse, 2018).

##### 3.2.2.7 Mortality

The highest levels of mortality were observed in both working and non-working single mothers (McKetta et al., 2018, Sabbath et al., 2015), working single childless women (McKetta et al., 2018) and in non-working married mothers (Sabbath et al., 2015). The lowest levels of mortality were observed in non-working married women who had children later (McKetta et al., 2018) and married women who took a break after childbirth and then returned to work (Sabbath et al., 2015). Differences in mortality rates between work-family trajectories were mostly explained by age, the number of births, race, and educational attainment (McKetta et al., 2018) and partially by smoking, alcohol consumption, BMI and household wealth in later adulthood (Sabbath et al., 2015). It remains unclear whether the protective effect on mortality can be explained by marital status or employment status. McKetta et al. (2018) emphasised the protective effect of marriage, whereas Sabbath et al. (2015) concluded that work is a protective factor for mortality regardless of partnership and parenthood status.

## 4 Discussion

This systematic review summarised work-family trajectories of men and women from different cohorts and countries and synthesised the observed associations between the trajectories and health. In recognition of the inter-relatedness of work and family domains across the life course, studies that analysed associations between work and family trajectories were included. Studying work and family trajectories simultaneously can help us to better understand the interplay between the two domains. The findings of the included studies highlight the benefits of applying a holistic approach, especially when examining the association with health. For example, the joint effect of the long-term unemployment and long-term absence of a partner on self-rated health is greater than the single effect of long-term unemployment or long-term absence of a partner (McDonough et al., 2015).

Work-family trajectories of men and women differed considerably; trajectories of women were found to be more diverse compared with men, i.e. the between-person differences were higher among women. Women’s trajectories were also more complex, i.e. the within-person differences were higher, with more transitions in their work trajectories (e.g. moving in and out of work, moving between full- and part-time employment). Trajectories of men and women became more similar in younger cohorts. For example, in people born between 1980 and 1984, the trajectories characterised by stable employment were as common in women as in men (Mooyaart et al., 2019), while in older age cohorts, women’s trajectories were less frequently characterised by stable employment compared with men (e.g. Vidal et al., 2020). Furthermore, women from younger cohorts took shorter and fewer breaks from paid employment, were less often stay-at-home mothers, and more often postponed parenthood compared with women in older cohorts. Men’s trajectories became more complex in younger cohorts, as unemployment and switching partners became more common. In some studies, weaker ties to employment became more prevalent in younger cohorts of men (e.g. Scherger et al., 2016), but overall, the most common work-family trajectory in men remained predominated by full-time employment. McMunn et al. (2015) were able to compare work-family trajectories of cohorts born in 1946, 1958 and 1970 and also observed the convergence of trajectories of men and women in the youngest cohort. This was mainly due to women being employed more consistently over time rather than men adjusting their work-family trajectories. England (2010) argues that women’s increased employment has not been reciprocated by changes in men’s family lives. Further, many work practices, such as hiring, training or promotion, are designed within the context of traditional gender role division where, for instance, women tend to reduce their work hours after giving birth (Moen & Sweet, 2004). According to Goldscheider, Bernhardt, and Lappegård (2015), the process of men’s increasing participation in private spheres is well underway in several countries, which is illustrated by young men’s more accepting attitudes towards gender equality. However, the findings of McMunn et al. (2015) do not support this shift as they found that work-family trajectories of men from older and younger age cohorts remained similar. Thus, the transition to parenthood seems to remain the critical point for the development of a gender gap in time spent on work and family care (e.g. Baxter, Hewitt, & Haynes, 2008; Kühhirt, 2012; Schober, 2013). The results of two studies (Jin et al., 2020; Mooyaart et al., 2019) differed from the other included studies. Specifically, these two studies did not find differences between the trajectories of men and women, which can be explained in several ways. The study by Jin et al. (2020) analysed a sample of people who were disproportionately highly educated and had a higher income than the general population. It is known from the literature that education shapes attitudes towards gender roles, suggesting that differences in attitudes towards gender roles attenuate when the educational level is higher (Deole & Zeydanli, 2021). The sample in the study by Mooyaart et al. (2019) was relatively young with people born between 1980 and 1984. It has been shown that trajectories of men and women are becoming more similar in younger cohorts (McMunn et al., 2015). In our review, only a few studies have focused on this youngest age cohort currently entering the labour market, and more research with longer follow-up and detailed data on both employment and family formation is needed to examine whether and how trajectories between men and women may further converge in this group. This is especially relevant given the context of changing employment patterns and growing precarious employment (Benach, Vives, Tarafa, Delclos, & Muntaner, 2016) that inevitably affects people’s life choices.

We have compared work-family trajectories in different geographical contexts as the different employment, family and work-family policies can influence the timing and ordering of education, employment and family formation processes. Many previous studies examined the impact of various work-family policies for combining work and family lives and showed that countries can influence work and family outcomes in both men and women through various interventions e.g. providing parental leave, childcare, child benefits or flexible work policies (Cukrowska-Torzewska, 2017, Hegewisch and Gornick, 2011, Misra et al., 2011). In our review, most studies examined work-family trajectories among US and European populations. Differences between the US and European countries were observed across studies and mainly pertained to women’s trajectories. Specifically, work-family trajectories of US women were more similar to those of men and were more often characterised by full-time employment in comparison with European women. Women’s working lives are heavily influenced by work-family policies, e.g. the length of maternity leave (Hegewisch & Gornick, 2011). For example, the UK historically supported a traditional division of gender roles that resulted in a high percentage of women working part-time (McDonough et al., 2015). In contrast, the US provide the shortest maternity leave out of all high-income countries (OECD, 2019), which may explain the observed higher involvement in continuous full-time employment compared with UK women (McDonough et al., 2015). There is a need for more comparative studies to further elucidate how contextual factors, for example, parental leave and work-family policies, influence work-family trajectories.

Our second research aim was to examine the association between identified work-family trajectories and health. Almost half of the included studies examined the association between work-family trajectories and health, and a significant association between trajectories and health was observed in almost all studies. The studies were diverse in the type of health variables included and the analytical approach. A rigorous synthesis of the evidence on the association between work-family trajectories and health was therefore hampered, and the results need to be interpreted within the broader context of each study. For example, Amato and Kane (2011) found that among women, respondents with more depressive symptoms in adolescence were more likely to belong to trajectories characterised by single motherhood, while Salmela-Aro et al. (2014) showed that respondents with more depressive symptoms were more likely to belong to trajectories *slow career starters* and *singles with slow careers* (i.e. postponed trajectories). This difference may be explained by the different study populations: Amato and Kane (2011) included a sample of the US general population, Salmela-Aro et al. (2014) included a selective sample of Finnish first-year university students. We have provided a synthesis of health variables that were examined in multiple studies, suggesting that some particular characteristics of work-family trajectories, i.e. an early transition to parenthood, single parenthood, and weak ties to employment seemed to be consistently associated with worse health across studies. In contrast, better health was found in people who stayed in education longer, were continuously working and postponed parenthood. Multiple studies showed that women who took a break from employment after childbirth, or temporarily switched to part-time work, had better health outcomes.

It is important to emphasise the diversity of the included studies regarding the historical context and country context, characteristics of the analysed sample (i.e. sex, life stage), variability in the health measurement, methodological considerations and other contextual factors. One of the major distinguishing factors in the analyses of work-family trajectories and their association with health was the life stage of interest. Differential effects of work and family events in different stages of adulthood have been described. For example, some researchers suggest that unemployment in young adulthood is especially harmful to health, with consequences of youth unemployment persisting till middle age (e.g. Strandh, Winefield, Nilsson, & Hammarström, 2014). Other scholars point out that older people have a higher chance of developing mental health problems when they are utnemployed than younger people (Woo & Zhang, 2020). The differential effects of life events at different life stages can be illustrated by our synthesis of the association between work-family trajectories and obesity. Lacey et al. (2017) analysed work-family trajectories from adolescence till late adulthood, while Mooyaart et al. (2019) constructed trajectories for the period of young adulthood only. Lacey et al. (2017) showed that early parenthood followed by longer spells of unemployment in midlife is associated with a higher risk of obesity, whereas Mooyaart et al. (2019) showed that in young adulthood, combining work and parenthood was associated with a higher risk of obesity. However, these two studies also differed in other important aspects, e.g. birth cohort and related socio-political context in which people build their work and family lives. In short, a detailed description of the sample recruitment and characteristics, contextual factors, as well as the life stage of interest, is paramount when interpreting the findings on the relationship between work, family and health.

Two main theories on how combining work and family affects health have been proposed: people either feel strain due to combining multiple roles, i.e. a conflict theory (Greenhaus & Beutell, 1985), or people benefit from combining work and family, i.e. a theory of role accumulation (Sieber, 1974). The included studies of this review found evidence for both theories, potentially due to their heterogeneity. For example, consistent with conflict theory, higher levels of depression were observed in women who returned to work full-time rather than part-time after parental leave (Engels et al., 2019). However, some studies in this review found better health outcomes in people in trajectories characterised by combining paid work and family roles in comparison with people in other trajectories, e.g. women in the trajectory *married mother full-timer* had a lower risk of depression and better self-rated health compared with *single at-home mothers* and *married at-home mothers* (McDonough et al., 2015). Previous studies also found mixed results; either a decline in the health benefit of employment when it was combined with childcare (Hewitt et al., 2006, Schnittker, 2007) or better health outcomes when occupying multiple roles, e.g. better well-being (Ahrens & Ryff, 2006) or good self-rated health (Fokkema, 2002, Janzen and Muhajarine, 2003, Kostiainen et al., 2009). As both conflict theory and theory of role accumulation have been supported by empirical evidence, the main challenge for future research is to elucidate why and under which conditions certain work-family trajectories are associated with either worse or better health. Also, several of the included studies emphasised the importance of examining further mechanisms through which work-family trajectories influence later health. Suggested mechanisms ranged from the physiological response to social stressors such as early parenthood (Lacey, Kumari et al., 2016) to the growing income gap as a consequence of motherhood, and a lack of social support due to single status (McKetta et al., 2018). Regarding the work domain, weaker ties to employment or low income were shown to have long-lasting health consequences for later life. For example, work-family trajectories characterised by part-time work were associated with better cognitive health possibly due to cognitive stimulation of employment and the absence of work-family conflict (Ice et al., 2020). For a more rigorous investigation of how and why combining different work and family roles affects and is affected by health, more research that examines the underlying mechanisms in different contexts is needed.

### 4.1 Strengths and limitations

This review is the first rigorous systematic synthesis of the literature on work-family trajectories and the association between work-family trajectories and health. The strengths of the review are the use of the systematic approach, the assessment of the risk of bias of each included study by two authors and a literature search not restricted to the English language or year of publication. There are also some limitations to our review. First, a few relevant search terms had to be excluded in the abstract search because the terms had too many different, irrelevant meanings, such as the term “work”. Nevertheless, the search strategy remained broad with a great diversity of words used for describing work and family trajectories and resulting in over 11,000 unique references. To lower the risk of missing relevant articles through our decision of excluding highly sensitive words in the abstract search, we searched the references of included articles and checked the publications of primary authors. Second, to assess the risk of bias, we could not use one of the existing quality checklists. This was due to the diverse study designs of the included studies and the lack of existing checklists addressing the risk of bias issues specifically relevant for studies applying sequence analysis and latent trajectory analysis. Hence, we combined items of existing checklists and added items relating to the quality of the studies constructing trajectories. Given the increasing use of sequence analysis to investigate life course trajectories, it may be worthwhile to develop the risk of bias items specifically related to this type of analysis as well as a checklist for assessing the quality of reporting in sequence analysis, such as the GRoLTS checklist for reporting on latent trajectory studies (van de Schoot, Sijbrandij, Winter, Depaoli, & Vermunt, 2017).

### 4.2 Recommendations for future research

The majority of the included studies analysed samples from Western countries and only a few studies focused on younger generations. To increase generalisability, we recommend focusing on the trajectories of people from other countries. Furthermore, the cohorts of current young adults are building their lives in times of turbulent societal changes and labour market challenges (e.g. labour market insecurity) and it will be important to investigate how this affects young people’s choices regarding work and family lives (Benach et al., 2016). Currently, there are only two studies focusing on the work-family trajectories of people born after 1981 (Bennett and Waterhouse, 2018, Mooyaart et al., 2019). The absence of the evidence on work-family lives of young people stems from the lack of longitudinal population cohort and panel studies in younger generations. Future research needs high-quality cohort data on younger cohorts. Additionally, studies that compare samples from different cohorts or countries might help to shed light on the role of contextual factors (e.g. different policies) on work-family trajectories.

Health was mostly assessed at one time point either as antecedent or consequence of the trajectory. We recommend assessing health repeatedly over time, as some of the included studies did (e.g. Carmichael & Ercolani, 2016; Lacey et al., 2017; Oesterle et al., 2011). These studies showed that in some cases, health differences observed after the assessment of work-family trajectories were already present before the start of these trajectories. In future studies, it is necessary to include markers of prior health in the analytical models to address potential reverse causation. In line with the principles of the life-course approach, knowledge is needed on how health in early life may select people into certain work-family trajectories and how it continues to affect future health (Amick et al., 2016, Kuh et al., 2003).

Lastly, we recommend rigorous reporting on the representativeness of the analysed samples. More than half of the included studies were rated as having a high risk of selection bias and it was not clear how selective the studied populations were. A rigorous reporting should include a detailed description of the analytical sample size, characteristics of the non-respondents and the approach of handling missing data.

## 5 Conclusion

This review summarised the evidence from studies analysing work-family trajectories, providing a detailed summary of work-family trajectories of men and women from different age cohorts and contexts, and a synthesis of the evidence on the association between work-family trajectories and health. Work-family trajectories differed greatly for men and women, but the differences seemed to decrease in the youngest cohorts. Given the current rapid changes in the labour market and work contexts, as well as changes to the gendered division of family care, it is important to investigate the work and family lives of the current generation of young adults. More comparative research could provide better insight into the role of the labour market and family policies on work and family decisions. Finally, work-family trajectories were found to be associated with health at different life stages. Future research should examine the longitudinal association of work-family trajectories with health and focus on elucidating why and under which circumstances some trajectories are associated with better or worse health compared with other trajectories.

## Funding

This paper is funded as part of a Netherlands Organization for Scientific Research (NWO) Vici project (’Today’s youth is tomorrow’s workforce: Generation Y at work’; NWO Vici 453-16-007/2735) that was granted to UB.

## Figures and Tables

**Table:** 

**1. SELECTION BIAS – This domain addresses whether the study sample is representative of the population of interest.**	

**Issues to consider**	**Description of an item**
*Source of target population*	The study population is adequately described, including who the target population is regarding sex and age and context (time period of the study and location)
*Method used to identify population*	The sampling frame and recruitment process for the study are adequately described
*Recruitment period*	Period of recruitment is adequately described
*Place of recruitment*	Place of recruitment (setting and geographic location) is adequately described
*Inclusion and exclusion criteria*	Inclusion and exclusion criteria are adequately described
*Adequate study participation*	There is adequate participation in the study by eligible individuals
*Baseline characteristics*	The baseline characteristics of the sample selected for trajectories analysis are adequately described, the description in the text is sufficient. Comprehensive description would include characteristics of age, sex, socioeconomic status, education, work (employment status, hours, or type of employment), family (union formation, having children) and health outcome of interest
**LOW RISK OF BIAS**	The study sample represents the population of interest on key characteristics, participation rate is adequate, all aspects of the recruitment process are well described or the authors reference to the description previously published elsewhere, and baseline sample is well described
**MODERATE RISK OF BIAS**	The study sample does not represent the general population, or participation rate is not clear, or recruitment process is not well described, or baseline sample is not well described
**HIGH RISK OF BIAS**	There are more problematic aspects that could introduce bias into the study

**2. ATTRITION BIAS – This domain addresses whether participants completing the study (i.e. with follow-up data) represent the baseline sample.**	

**Issues to consider**	**Description of an item**
*Proportion of baseline sample available for analysis*	Response rate (i.e. proportion of respondents with complete longitudinal data that could be included in the trajectories analysis) is adequate
*Reasons and potential impact of subjects lost to follow-up*	Reasons for missing data and excluding respondents from trajectories analysis are provided
*Information on those lost to follow-up*	Participants that could not be included into the trajectories analysis because of an incomplete dataset were described for demographic characteristics, work, family and health condition (if it is a focus of the study)
**LOW RISK OF BIAS**	The authors of the study address the possible impact of loss to follow-up on the suggested typology of work and family trajectories. In case of low attrition, information is provided that missingness is convincingly at random
**MODERATE RISK OF BIAS**	Lower follow-up rates, or not providing reasons for loss to follow-up, or not reporting differences between dropouts and respondents with complete data, or significant differences between dropouts and respondents with complete data
**HIGH RISK OF BIAS**	There are more problematic aspects that could introduce bias into the study

**3. MEASUREMENT AND RECALL BIAS: WORK AND FAMILY – This domain addresses the adequate measurement of the work and family states**	

**Issues to consider**	**Description of an item**
*Definition of work and family states*	The possible work and family states are clearly defined and include all possible work and family situations relevant to the research question of interest
*Method of work and family measurement*	The method and setting of measurement of work and family states is the same for all study participants
*Recall bias*	The recall period was reasonable to recall major life events given the work and family states that need to be recollected
*Method used for missing data*	Appropriate methods of imputation are used for missing individual data on work and family. The following items are reported: amount of missing data, reasons for missingness, method for handling the data, assumptions that were made, number of imputed datasets and complete-case analysis
**LOW RISK OF BIAS**	Work and family states are clearly defined, the method of measurement/data collection is the same for all study participants, the data were collected longitudinally and the method of imputing missing data is appropriate and well described
**MODERATE RISK OF BIAS**	Work and family states are not clearly defined, or the method of measurement/data collection is not the same for all study participants, or the data might be influenced by the long recall period, or the method of imputing missing data is not appropriate and well described
**HIGH RISK OF BIAS**	There are more problematic aspects that could introduce bias into the study

**4. MEASUREMENT BIAS: HEALTH – This domain addresses adequacy of measurement of the health condition toward non-differential measurement related to work and family trajectories.**	

**Issues to consider**	**Description of an item**
*Definition of the health condition*	The health condition is clearly defined
*Valid and reliable measurement of health*	The health condition is measured by a valid and reliable scale
*Method of health outcomes measurement*	The method and setting of the measurement of the health condition is the same for all study participants
*Proportion of respondents with data on health*	The proportion of respondent with constructed trajectories who have complete health data is reported, reasons for missing data are reported, and information on respondents with incomplete health data provided
*Method used for missing data*	Appropriate methods of imputation are used for missing individual health data. The following items are reported: amount of missing data, reasons for missingness, method for handling the data, assumptions that were made, number of imputed datasets and complete-case analysis
**LOW RISK OF BIAS**	A clearly defined health condition is measured by a valid and reliable scale and the data are collected the same way in every respondent. Missing data are handled adequately and differences between respondents with and without missing data are described
**MODERATE RISK OF BIAS**	The health condition is not clearly defined or it is not measured by a valid and reliable scale, or the data are not collected the same way in every respondent, or missing data were not handled adequately, or differences between respondents with and without missing data are not described
**HIGH RISK OF BIAS**	There are more problematic aspects that could introduce bias into the study

**5. STUDY CONFOUNDING – This domain addresses potential confounding, or distortion of the relationship between the health condition and work-family trajectories by another factor**	

**Issues to consider**	**Description of an item**
*Reasons for selecting confounders*	The reasons for selecting specific confounders are explained
*Definition of the confounding factor*	Clear definitions of the important confounders are provided
*Method used for missing data*	Appropriate methods are used if imputation is used for missing confounder data. The following items are reported: amount of missing data, reasons for missingness, method for handling the data, assumptions that were made, number of imputed datasets and complete-case analysis
*Appropriate accounting for confounding*	Important potential confounders are accounted for in the analysis (i.e. appropriate adjustment)
*Prior health*	The authors have included an earlier indicator of the analysed health variable in the analysis of the association between work-family trajectories and subsequent health, e.g. by adjusting for earlier health, or by selecting the sample with no prior health problems
**LOW RISK OF BIAS**	The definition of relevant confounders is provided and in case of missing data, an appropriate method is used for imputation. Important confounders are accounted for in the analysis. Potential confounders include gender, context (cohort, country), socioeconomic status, education and other measured health conditions. In studies that analysed health as a consequence of work-family trajectories, the prior health was considered in the analysis.
**MODERATE RISK OF BIAS**	Confounders are not clearly defined, or missing data were not appropriately handled, or the possible reverse causation was not taken into account.
**HIGH RISK OF BIAS**	There are relevant confounders that were not accounted for in the analysis. Confounding was not considered. Reverse causation was not accounted for.

**6. STATISTICAL ANALYSIS AND REPORTING – This domain addresses the appropriateness of the study’s statistical analysis and completeness of reporting.**	

**Issues to consider**	**Description of an item**
*Presentation of analytical strategy (why)*	The authors explain why they used a selected analytical method (e.g. they explain why they applied a sequence analysis or why they used a specific clustering method). The selected method is suitable for the specific research question
*Model development strategy (how)*	They report on an analytical strategy to build trajectories and create clusters/typology
*Reporting of results*	There is no selective reporting of results
**LOW RISK OF BIAS**	The reasons for selecting a specific method are well explained and all the steps that authors undertook are well described. There is no selective reporting of results
**MODERATE RISK OF BIAS**	The authors do not explain why they used the selected statistical method or they do not provide the details on how the analysis was conducted or they do not report results for all the groups of interest
**HIGH RISK OF BIAS**	There are more problematic aspects that could introduce bias into the study

**Table:** 

	**Aim**	**Source of the data**	**Country**	**Study design**	**N**	**Year of birth**	**Trajectories in age range**	**Women (%)**	**Statistical method**	**Unit of the trajectory**	**Variables**
Aassve et al. (2007)	to study young women's trajectories in Great Britain	British Household Panel Survey	UK	longitudinal study (combined with 1992 retrospective survey)	578	1960–1969	13–30	100	sequence analysis	month	1) employment 2) parenthood 3) partnership
Aeby et al. (2019)	to investigate the impact of whole work-family trajectories on the composition of personal networks	Family tiMes	Switzerland	retrospective survey	382	1970–1975	start between 16–21; end between 36–41	53.0	sequence analysis	semester (two periods per year)	1) employment 2) residency incl. parenthood and partnership
Aisenbrey and Fasang (2017)	to examine how gender inequality in work-family trajectories unfolds from early adulthood until middle age	National Longitudinal Survey of Youth German National Education Panel Study	US, Germany	longitudinal study (interviews every year, from 1994 every two years) retrospective survey	8630	1957–1964 1956–1965	22–44	not reported	sequence analysis	month	1) employment, incl. education 2) combined parenthood and partnership
Amato et al. (2008)	to describe most common pathways in young women and explore their predictors	National Longitudinal Study of Adolescent Health	US	longitudinal study (but only data from third wave were used to construct trajectories)	2290	1976–1979	18–23	100	LCA	year	1) education 2) employment 3) cohabitation 4) marital status 5) parenthood
Amato and Kane (2011)	to examine early life-course pathways and their links with general health and psychosocial adjustment	National Longitudinal Study of Adolescent Health	US	longitudinal study (but only retrospective data from third wave were used to construct trajectories)	2290	1976–1979	18–23	100	LCA	year	1) education 2) employment 3) cohabitation 4) marital status 5) parenthood
Arpino et al. (2018)	to examine to what extent the effect of early-life conditions (health and socioeconomic status) on health in later life is mediated by educational attainment and life-course trajectories (fertility, partnership, employment)	Survey of Health, Ageing and Retirement in Europe	SHARE countries	longitudinal study (but only retrospective data from third wave were used to construct trajectories)	12,034	before 1949	15–59	51.7	sequence analysis	year	1) employment 2) parenthood 3) partnership
Barnett (2013)	to investigate pathways of adult child caregivers’ family (caregiving, marital, parenting) and nonfamily (employment) roles	Health and Retirement Study	US	longitudinal study (8 waves)	1300	1931–1941	start between 51–61; end between 65–75	79.0	LCA	wave (8 waves across 14 years)	1) employment 2) parenthood 3) marital status 4) caregiving
Bennett and Waterhouse (2018)	to analyse patterning of key transitions and the association between different pathways and health	National Income Dynamics Survey	South Africa	longitudinal study (4 waves)	429	1991–1993	start between 15–17; end between 21–24	100	sequence analysis	wave (4 waves across 7 years)	1) employment, incl. education 2) parenthood 3) partnership
Carmichael and Ercolani (2016)	to investigate the extent to which people's earlier circumstances and experiences shape subsequent life-courses and to see whether the differences between clusters remain the same over time	British Household Panel Survey and follow-on Understanding Society	UK	longitudinal study (18 waves of BHPS and subsequent US measurements)	4339	1906–1980	16–85 at the beginning of the trajectory, followed for 15–20 years	56.0	sequence analysis	year	1) employment, incl. education 2) parenthood 3) caregiving
Comolli et al. (2021)	to investigate whether and how employment and family trajectories are jointly associated with subjective, relational and financial wellbeing later in life	Swiss Household Panel	Switzerland	retrospective survey	1885	1933–1966	20–50	53.3	sequence analysis	year	1) employment 2) parenthood 3) partnership
Davia and Legazpe (2014)	to describe the evolution of labour market participation and fertility patterns	Spanish Fertility, Family and Values Survey of 2006	Spain	retrospective survey	1946	1956–1970	16–35	100	sequence analysis	year	1) employment 2) parenthood 3) partnership
Engels et al. (2019)	to identify types of work-family trajectories of men and women and to investigate their links with depression at older age	Heinz Nixdorf Recall study	Germany	longitudinal study (but only data from second wave were used to construct trajectories)	3019	1925–1955	20–50	50.9	sequence analysis	year	1) employment 2) parenthood
Huang et al. (2007)	to identify women’s career development patterns by examining the dynamic interactions between individuals’ involvement in working life and other career-related domains of life	Individual Development and Adaptation	Sweden	longitudinal study (but only retrospective data from one wave were used to construct trajectories)	549	1955	16–43	100	sequence analysis	6 months	1) combined employment and education incl. parental leave
Ice et al. (2020)	to investigate the relationship between women’s work-family life histories and cognitive functioning in later life	Survey of Health, Ageing and Retirement in Europe	14 SHARE countries^1^	longitudinal study (but only retrospective data from third wave were used to construct trajectories)	11,908	1930–1957	12–50	100	sequence analysis	year	1) employment, incl. education and caregiving 2) combined parenthood and partnership
Jin et al. (2020)	to investigate the heterogeneous effects of life events on travel mode use and further differentiation between gender and generation groups in these life event effects	WholeTraveler Trans- portation Behavior Study	US	retrospective survey	569	1930–1983	20–35	49.0	sequence analysis	year	1) education 2) employment 3) partnership 4) parenthood
Johansson et al. (2007)	to investigate if and how health and well-being in mid-life are influenced by the ways in which individuals have combined educational, occupational, and family involvement throughout their adult lives	Individual Development and Adaptation	Sweden	longitudinal study (but only retrospective data from one wave were used to construct trajectories)	549	1955	16–43	100	sequence analysis	6 months	1) combined employment and education incl. parental leave
Koelet et al. (2015)	to examine the diverse ways in which young adults develop both their professional career and family life	SONAR	Belgium	longitudinal study (3 waves supplemented by retrospective survey at each wave)	1598	1976	14–29	49.6	sequence analysis	month	1) employment, incl. education 2) parenthood 3) partnership
Lacey, Kumari et al. (2016)	to investigate whether the combined work-family life courses of British men and women were associated with differences in metabolic markers	National Survey of Health and Development	UK	longitudinal study (23 waves, 10 waves used to construct trajectories)	2503	1946	16–51	50.0	sequence analysis	year	1) employment 2) parenthood 3) partnership
Lacey, Sacker et al. (2016)	to investigate associations between work-family life courses and biomarkers of inflammation and stress	National Child Development Study	UK	longitudinal study (10 waves, not clear how many waves were used to construct trajectories)	7228	1958	16–42	51.1	sequence analysis	year	1) employment 2) parenthood 3) partnership
Lacey, Stafford et al. (2016)	to characterise work-family life courses across adulthood and assess its link to subjective well-being	National Survey of Health and Development	UK	longitudinal study (21 waves, not clear how many waves were used to construct trajectories)	2000	1946	16–60	not reported	sequence analysis	year	1) employment 2) parenthood 3) partnership
Lacey et al. (2017)	to investigate relationships between work-family life courses and BMI trajectories across adulthood	National Survey of Health and Development National Child Development Study British Cohort Study	UK	longitudinal studies (21 waves, 11 waves, 8 waves, not reported which waves were used to build trajectories)	20,760	1946 1958 1970	16–42	1946: 50.6 1958: 51.3 1970: 53.3	sequence analysis	year	1) employment 2) parenthood 3) partnership
Madero-Cabib and Fasang (2016)	to examine the association between gendered work–family life courses and financial well-being in retirement	Survey of Health, Ageing and Retirement in Europe	Switzerland, Germany	longitudinal study (but only retrospective data from third wave were used to construct trajectories)	1709	1920–1950	20–59	55.1 48.8	sequence analysis	year	1) employment 2) parenthood 3) partnership
Madero-Cabib et al. (2016)	to assess the link between work and family experiences and timing of retirement	Survey of Health, Ageing and Retirement in Europe	Switzerland	longitudinal study (but only retrospective data from third wave were used to construct trajectories)	674	before 1951	20–57	41.1	sequence analysis	year	1) employment combined with pension investments 2) parenthood combined with marital status
Mayeda et al. (2020)	to examine whether life course patterns of employment, marriage, and childrearing influence later-life rate of memory decline among women	HRS	US	longitudinal study (not reported which waves were used to build trajectories)	6189	1935–1956	16–50	100	sequence analysis	year	1) employment 2) parenthood 3) partnership
McDonough et al. (2015)	to investigate whether adverse circumstances early in the life course cumulate as health-harming biographical patterns across the prime working and family caregiving years	National Longitudinal Survey of Youth National Childhood Development Study	US, UK	longitudinal study (annual and later biannual waves) longitudinal study (10 waves) data from all waves in which the respondents were 25–49 years were used	8455	1957–1964 (US) 1958 (UK)	25–39	100	sequence analysis	year	1) employment 2) marital status
McKetta et al. (2018)	to assess the association between work-family trajectories and mortality rate	Panel Study on Income Dynamics	US	longitudinal study (trajectories based on data from 1968 to 2013 waves)	6039	1927–1978	18–50	100	sequence analysis	year	1) employment 2) parenthood 3) partnership
McMunn et al. (2015)	to examine whether there is increase in between-person de-standardization and within-person differentiation in life courses across cohorts, to investigate whether men's and women's life courses are converging, to assess the link between education and life courses	National Survey of Health and Development National Child Development Study British Cohort Study	UK	longitudinal studies (not reported which waves were used to build trajectories)	20,786	1946 1958 1970	16–42	1946: 50.6 1958: 51.3 1970: 53.3	sequence analysis	year	1) employment 2) parenthood 3) partnership
McMunn et al. (2016)	to investigate associations between work-family life course types (LCTs) and markers of metabolic risk	National Child Development Study	UK	longitudinal study (10 waves, not clear which waves were used to build trajectories)	7228	1958	16–42	51.1	sequence analysis	year	1) employment 2) parenthood 3) partnership
Mooyaart et al. (2019)	to examine what career and family life-course pathways during the transition to adulthood are related to developing obesity in young adulthood	National Longitudinal Survey of Youth	US	longitudinal study (data collected yearly)	4688	1980–1984	17–27	52.9	sequence analysis	month	1) combined employment and education 2) combined parenthood and partnership
Müller et al. (2012)	to assess life trajectories of individuals with psychiatric problems	systematic data collection in 2 distinct facilities	Switzerland	retrospective survey	86	not reported	occupation and co-residency: 18+; intimacy: 16+; up till the current age or 34	49.0	sequence analysis	year	1) employment, incl. education 2) partnership 3) residency incl. parenthood and partnership
Mynarska et al. (2015)	to reconstruct the major life course trajectories of childless women and reveal the complexity of the life paths that lead to childlessness	Household Multipurpose Survey on Family and Social Subjects FAMWELL Survey on Childlessness	Italy, Poland	retrospective surveys	920	1965–1974	15–37	100	sequence analysis	month	1) employment 2) education 3) partnership
Oesterle et al. (2010)	to examine how commonly observed pathways to adulthood, defined by education, employment, marriage, and parenthood, were associated with alcohol, tobacco, and marijuana misuse	Seattle Social Development Project	US	longitudinal (data from 5 waves used to construct trajectories)	728	1975	18–30	50.3	LCA	wave (5 waves across 12 years)	1) employment 2) education 3) parenthood 4) partnership
Oesterle et al. (2011)	to examine how commonly observed pathways to adulthood, defined by education, employment, marriage, and parenthood, were associated with alcohol, tobacco, and marijuana misuse	Seattle Social Development Project	US	longitudinal (data from 5 waves used to construct trajectories)	728	1975	18–30	50.3	LCA	wave (5 waves across 12 years)	1) employment 2) education 3) parenthood 4) partnership
Pailhé (2013)	to investigate the degree of interaction between work and family of both partners in the long run over the life course	Enquête Familles et Employeurs (Families and Employers Survey)	France	retrospective survey	941 couples	1954–1968	start between 18–31; end between 36–49	NA	sequence analysis	year	1) employment incl. education 2) parenthood
Piccarreta and Billari (2007)	to construct cluster of life course trajectories and to obtain ideal types of trajectories	British Household Panel Survey	UK	longitudinal study (not reported how many waves were used to build trajectories)	578	1960–1968	13–30	100	sequence analysis	month	1) employment 2) parenthood 3) partnership
Pollock (2007)	to demonstrate the use of sequence analysis the examination of multivariable combinations of status as they change over time	British Household Panel Survey	UK	longitudinal study (waves between 1991 and 2000 used to build trajectories)	5124	not reported	all respondents analysed in calendar years 1991–2000	not reported	sequence analysis	year	1) employment incl. education 2) parenthood 3) marital status 4) housing tenure
Sabbath et al. (2015)	to examine relationships between US women’s exposure to midlife work–family demands and subsequent mortality risk	Health and Retirement Study	US	longitudinal study (not reported how many waves were used to build trajectories)	7536	1936–1956	16–50	100	sequence analysis	year	1) employment 2) parenthood 3) partnership
Salmela-Aro et al. (2011)	to examine the sequences, patterns, and variations in family and work-related roles during the transition to adulthood among university students	Helsinki Longitudinal Student Study	Finland	retrospective survey	182	1966–1973	start between 18–25; end between 36–43	78.0	sequence analysis	year	1) employment, incl. education 2) parenthood 3) partnership
Salmela-Aro et al. (2014)	to examine the extent to which achievement and social strategies, and depressive symptoms contribute to pathways to adulthood	Helsinki Longitudinal Student Study	Finland	retrospective survey	182	1966–1973	start between 18–25; end between 36–43	78.0	sequence analysis	year	1) employment, incl. education 2) parenthood 3) partnership
Scherger et al. (2016)	to assess the extent to which individuals’ life course trajectories vary across cohorts, gender, and level of education	English Longitudinal Study of Ageing	UK	longitudinal study (retrospective life history calendar in wave 3)	6334	1916–27 1928–37 1938–47 1948–57	15–50	52.7	sequence analysis	year	1) employment 2) parenthood 3) marital status
Sirniö et al. (2017)	to describe the most typical work-family trajectories and to assess whether belonging to these trajectories is associated with parental origin	Register-based data	Finland	longitudinal study based on register data	23,915	1972–1975	16–37	48.5	sequence analysis	year	1) employment, incl. education 2) combined parenthood, partnership and residency
Stafford et al. (2019)	to examine paid work at age 60–64 (and age 68–69 for men) by work–family patterns across 35 years	National Survey of Health and Development	UK	longitudinal study (data collected in interviews from age 16–51)	2513	1946	16–51	50.0	sequence analysis	year	1) employment 2) parenthood 3) partnership
Tocchioni (2018)	to identify typical life course trajectories of childless women and men in Italy from a gender perspective and in a cross-cohort comparison	Multipurpose Household Survey on Family and Social Subjects	Italy	retrospective survey	3414	1907–1969	16–50 (the trajectory is shorter for people who were 40–50 at the time of survey)	49.4	sequence analysis	month	1) employment 2) education 3) partnership
Van Hedel et al. (2016)	to investigate whether less-healthy work–family life histories contribute to the higher cardiovascular disease prevalence in older American compared with European women	Health and Retirement Study Survey of Health, Ageing and Retirement in Europe	US, 13 SHARE countries^2^	longitudinal study (retrospective data and self-reports from 1992 to 2006 wave were used to construct trajectories) longitudinal study (but only retrospective data from third wave were used to construct trajectories)	18,250	1935–1956	16–50	100	sequence analysis	year	1) employment 2) parenthood 3) partnership
Vidal et al. (2020)	to examine similarities across mothers’ and daughters’ work-family trajectories and to examine systematic associations between work-family trajectories	German Socio-Economic Panel	Germany	longitudinal study (not reported which waves were used to build trajectories)	1246	1930–1949 1958–1981	18–35	100	sequence analysis	year	1) combined employment and parenthood
Worts et al. (2013)	to examine women’s evolving work and family biographies	National Longitudinal Survey of Women – Young Women National Longitudinal Survey of Youth	US	longitudinal study (22 waves) longitudinal study (23 waves till 2008 when the analysis was conducted) data from all waves in which the respondents were 25–49 years were used	7150	1942–1945 1946–1949 1950–1953 1957– 1960 1961–1964	25–49	100	sequence analysis	year	1) employment 2) parenthood 3) partnership
Xue et al. (2020)	to investigate whether the timing and nature of women’s transitions out of full-time education are related to later-life subjective well-being and the life-course experiences that might explain any associations seen	English Longitudinal Study of Ageing	UK	retrospective survey	3889	before 1956	14–26	100	sequence analysis	year	1) employment, incl. education 2) parenthood 3) partnership
Zimmermann (2021)	to analyse the association between work-family life courses and life satisfaction	German Socio-Economic Panel	Germany	longitudinal study (not reported which waves were used to build trajectories)	2542	1920–1957	18–60	100	sequence analysis	year	1) employment 2) parenthood 3) partnership

UK, United Kingdom; US, United States LCA, latent class analysis

^1^SHARE countries: Austria, Belgium, Czech Republic, Denmark, France, Germany, Greece, Ireland, Italy, Netherlands, Poland, Spain, Sweden, and Switzerland

^2^SHARE countries: Austria, Belgium, Czech Republic, Denmark, France, Germany, Greece, Italy, Netherlands, Poland, Spain, Sweden, and Switzerland

**Table:** 

	**Year of birth**	**Country**	**Identified trajectories**	**% of women**			**% of men**			**% of the sample**
Aassve et al., (2007)*	1960-1969	UK	1) work-oriented, no family formation	8.7						
			2) education, late entry to work, late family formation	17.3						
			3) early work, early family formation without fertility	9.7						
			4) early work, late family formation without fertility	18.5						
			5) traditional: work, partnership, childbearing, exit labour market	15.2						
			6) short labour market attachment before family formation	10.7						
			7) propensity to high fertility with repeated job spells	10.7						
			8) earlier family formation and potential family instability	5.0						
			9) early work, child, dissolution of partnership, long period out of labour market, new partnership, second child	4.2						

Aeby et al., (2019)	1970-1975	Switzerland	1) double investment	8.5			41.4			
			2) family-focused	37.8			0.0			
			3) conciliation	16.4			9.9			
			4) conjugality/full-time	23.9			24.3			
			5) preparation	13.4			24.3			

Aisenbrey and Fasang (2017)	1957-1964	US, Germany	1) single, children, disrupted low prestige	20.0			12.0			
	1956-1965		2) couple, one child, medium prestige	13.0			19.0			
			3) couple, many children, medium prestige	17.0			16.0			
			4) couple, two children, medium prestige	26.0			23.0			
			5) single, childless, upward mobility	8.0			10.0			
			6) couple, childless, medium prestige	6.0			7.0			
			7) couple, children, highest prestige	11.0			12.0			

Amato et al., (2008)	1976-1979	US	1) college to job with no family	28.8						
			2) high school to job with no family formation	18.6						
			3) cohabiting without children	14.5						
			4) married mother	14.2						
			5) single mothers	9.7						
			6) cohabiting mothers	8.3						
			7) inactive	5.9						

Amato and Kane (2011)	1976-1979	US	1) college to job with no family	28.8						
			2) high school to job with no family formation	18.6						
			3) cohabiting without children	14.5						
			4) married mother	14.2						
			5) single mothers	9.7						
			6) cohabiting mothers	8.3						
			7) inactive	5.9						

Arpino et al., (2018)	before 1949	SHARE countries	**Women**							
			1) no union, inactive	1.9						
			2) children 3+, inactive	16.4						
			3) children 3+, married	12.3						
			4) two children, married	18.0						
			5) one child, married	10.1						
			6) children, no union	4.7						
			7) no children, married, employed	3.6						
			8) one child, inactive	4.8						
			9) two children, inactive	15.2						
			10) children 3+, part-time	4.9						
			11) no children, married, inactive	2.5						
			12) two children, part-time	5.5						

			**Men**							
			1) children 3+				30.8			
			2) no children, no union				4.4			
			3) no children, married				9.9			
			4) one child, married				13.2			
			5) two children, married				35.0			
6) low employment				2.1			

Barnett (2013)	1931-1941	US	1) not-married, early-transition to not-working caregivers							34.0
			2) married, not-working caregivers							26.0
			3) married, late-transition to not-working caregivers							23.0
			4) married, not-working caregivers with coresiding child							17.0

Bennett and Waterhouse (2018)	1991-1993	South Africa	1) non-activity commonly followed by motherhood	11.0						
			2) pathway from school, motherhood then work	29.0						
			3) motherhood combined with schooling	15.0						
			4) motherhood after schooling	21.0						
			5) schooling to non-activity	24.0						

Carmichael and Ercolani (2016)	1906-1980	UK	1) full-time careers							26.4
			2) evolving careers							23.2
			3) part-time careers							18.2
			4) caring intensive							10.2
			5) decaying careers							22.0

Comolli et al. (2021)	not reported, but between 1933-1966	Switzerland	**Women**							
			1) full-time work, traditional	6.5						
			2) full-time work, early traditional	8.0						
			3) full-time work, childless	6.9						
			4) return to part-time, traditional	20.8						
			5) return to part-time, early traditional	30.7						
			6) return to part-time, childless	2.5						
			7) not in employment, traditional	13.3						
			8) not in employment, early traditional	11.0						
			9) not in employment, childless	0.4						

			**Men**							
			1) early full-time work, traditional				38.0			
			2) early full-time work, late traditional				23.2			
			3) early full-time work, childless				15.5			
			4) full-time work after higher education, traditional				4.2			
			5) full-time work after higher education, late traditional				5.1			
			6) full-time work after higher education, childless				1.8			
			7) part-time work, traditional				4.7			
			8) part-time work, late traditional				3.6			
9) part-time work, childless				4.0			

Davia and Legazpe (2014)	1956-1970	Spain	1) early marriage/non-working mother/high fertility	22.0						
			2) late marriage/working mother/low fertility	39.0						
			3) early marriage/working mother/high fertility	11.2						
			4) late marriage/low participation/low fertility	27.8						

Engels et al., (2019)	1925-1955	Germany	**Women**							
			1) full-time work, no children	12.4						
			2) full-time work, children	15.7						
			3) re-entry in full-time work, children	11.7						
			4) re-entry in part-time work, children	32.1						
			5) part-time work, children	16.7						
			6) no work, early children	11.3						

			**Men**							
			1) full-time work, no children				17.9			
			2) full-time work, early children				22.3			
			3) full-time work, children				19.6			
			4) full-time work, late children				14.6			
			5) full-time work, many children				20.1			
6) unstable work, children				5.4			

Huang et al., (2007)	1955	Sweden	1) full timers	20.8						
			2) career oriented women	10.9						
			3) early mothers full time	8.4						
			4) working mothers	7.8						
			5) delayed family builders	14.2						
			6) early family builders	13.7						
			7) early mothers part-time	6.4						
			8) Scandinavian family builders	14.2						
			9) non-employed	3.6						

Ice et al., (2020)	1930-1957	SHARE countries	1) full-time working mothers	50.0						
			2) unpaid caregiver mothers	25.0						
			3) part-time working mother	12.0						
			4) single full-time working woman	9.0						
			5) mother with little unpaid care or paid work	4.0						

Jin et al., (2020)	1930-1983	US	1) singles	41.9			38.6			
			2) couples	26.5			26.6			
			3) have-it-alls	14.7			21.4			
			4) late bloomers	6.8			8.6			
			5) family first	9.0			5.9			

Johansson et al., (2007)	1955	Sweden	1) full timers	20.8						
			2) career oriented women	10.9						
			3) early mothers full time	8.4						
			4) working mothers	7.8						
			5) delayed family builders	14.2						
			6) early family builders	13.7						
			7) early mothers part-time	6.4						
			8) Scandinavian family builders	14.2						
			9) non-employed	3.6						

Koelet et al., (2015)	1976	Belgium	1) full-time worker, no family formation	18.0			37.0			
			2) full-time worker, initial labour market experience without family formation, eventually union formation, hardly any children	14.0			26.0			
			3) full-time worker, early union formation without children	14.0			13.0			
			4) full-time worker, early family formation with children	28.0			16.0			
			5) longer periods of part-time work, initially without family formation	14.0			7.0			
			6) mixed work and family strategies, early family formation with children	11.0			2.0			

Lacey, Kumari et al., (2016)	1946	UK	1) work, early family	15.1			47.7			
			2) work, marriage, non-parent	9.0			7.9			
			3) work, no family	6.1			11.5			
			4) work, later family	3.5			30.6			
			5) later family, work break	11.6			1.0			
			6) early family, work break	14.6			0.6			
			7) part-time work, early family	29.9			0.7			
			8) no paid work, early family	10.3			0.0			

Lacey, Sacker et al. (2016)	1958	UK	1) work, later family	8.9			34.4			
			2) work, cohabitation, later parent	5.1			6.5			
			3) work, marriage, non-parent	8.9			7.8			
			4) work, early family	11.7			31.9			
			5) later family, work break	14.0			0.2			
			6) work, no family	10.1			12.8			
			7) early family, work break	15.8			0.1			
			8) part-time work, early family	18.0			0.3			
			9) no paid work, early family	3.3			0.1			
			10) lone parent, divorced	2.5			4.2			
			11) teen parent	1.2			0.8			
			12) unstable work, no family	0.6			0.9			

Lacey, Stafford et al. (2016)	1946	UK	1) work, early family	6.9			33.9			
			2) work, early family, retired	10.7			16.2			
			3) work, later family	2.3			20.8			
			4) work, later family, retired	1.6			10.3			
			5) work, marriage, non-parent	7.0			7.8			
			6) work, no family	5.1			7.9			
			7) later family, work break	12.7			0.5			
			8) early family, work break	13.4			0.6			
			9) part-time work, early family	28.6			1.2			
			10) no paid work, early family	7.0			0.0			
			11) teen parent	4.6			0.9			

Lacey et al. (2017)	1946	UK		**1946**	**1958**	**1970**	**1946**	**1958**	**1970**	
	1958		1) work, no family	6.5	9.6	18.7	12.5	12.8	25.8	
	1970		2) work, marriage, non-parent	6.6	8.8	8.9	9.9	7.8	8.9	
			3) work, cohabitation, later parent	0.9	4.9	10.9	2.0	6.8	13.3	
			4) work, later family	3.0	8.6	11.4	28.7	33.9	29.7	
			5) work, early family	13.8	12.1	6.5	43.0	31.8	14.6	
			6) work, divorced parent	0.5	2.8	3.7	1.3	4.5	3.1	
			7) teen parent	2.5	1.3	3.0	0.4	0.2	0.5	
			8) later family, work break	14.1	13.1	12.3	0.4	0.2	0.5	
			9) early family, work break	17.3	16.2	6.7	0.0	0.1	0.1	
			10) part-time work, early family	21.4	18.1	12.9	0.3	0.3	0.2	
			11) no paid work, early family	12.5	4.1	3.2	0.0	0.1	0.2	
			12) unstable work, no family	0.9	0.6	2.0	1.5	1.1	2.8	

Madero-Cabib and Fasang (2016)	1920-1950	Switzerland, Germany	1) full-time employed, 2+ children	10.0			60.0			
			2) full-time employed, 1 child	4.0			18.0			
			3) out of the labor force, 2+ children	32.0			1.0			
			4) part-time employed, 2+ children	24.0			1.0			
			5) out of labor force or part-time employed, 1 child	15.0			1.0			
			6) full-time employed, divorce	5.0			5.0			
			7) full-time employed, childless married	7.0			9.0			
			8) full-time employed, childless unmarried	4.0			5.0			

Madero-Cabib et al. (2016)	before 1951	Switzerland	1) full-time worker, public and other pension funds / children family	4.8			45.3			
			2) out of the labor force & part-time worker, public and other pension funds / children family	60.4			4.4			
			3) full-time worker, only public pension funds / children family	6.2			24.3			
			4) full-time and part-time workers, only public pension funds / divorced	11.2			5.3			
			5) full-time worker, public and other pension funds / divorced	3.4			10.2			
			6) full-time worker, public and other pension funds / single	14.1			10.4			

Mayeda et al. (2020)	1935-1956	US	1) working nonmother	7.9						
			2) working married mother	69.9						
			3) working single mother	8.6						
			4) nonworking single mother	5.2						
			5) nonworking married mother	8.5						

McDonough et al. (2015)	1957-1964	US, UK		**US**	**UK**					
	1958		1) married mother full- timer	40.7	28.6					
			2) married mother part-timer	11.9	25.0					
			3) married at-home mother	22.3	23.0					
			4) divorcing back-to-work mother	3.0	4.0					
			5) single employed mother	14.9	13.0					
			6) single at-home mother	7.2	6.4					

McKetta et al. (2018)	1927-1978	US	1) non-working, married, later-mothers	37.8						
			2) working, divorced mothers	35.1						
			3) working and non-working never-married mothers	9.4						
			4) working, never-married non-mothers	5.6						
			5) non-working, married, earlier-mothers	12.0						

McMunn et al. (2015)	1946	UK		**1946**	**1958**	**1970**	**1946**	**1958**	**1970**	
	1958		1) work, no family	4.8	9.7	18.7	11.1	12.9	25.7	
	1970		2) work, marriage, non-parent	6.6	8.8	8.9	9.6	7.9	9.2	
			3) work, cohabitation, later parent	1.1	5.1	10.9	2.3	7.0	13.4	
			4) work, later family	2.7	8.5	11.4	26.1	33.1	29.7	
			5) work, early family	15.0	11.8	6.5	46.8	31.6	14.5	
			6) work, divorced parent	0.6	2.8	3.7	1.3	4.6	3.1	
			7) teen parent	3.0	1.5	2.9	0.5	0.9	0.5	
			8) later family, work break	12.5	13.1	12.3	0.6	0.2	0.5	
			9) early family, work break	17.1	16.1	6.6	0.0	0.1	0.1	
			10) part-time work, early family	22.6	17.6	12.9	0.4	0.3	0.2	
			11) no paid work, early family	13.4	4.3	3.2	0.0	0.1	0.2	
			12) unstable work, no family	0.6	0.6	2.0	1.4	1.2	2.8	

McMunn et al. (2016)	1958	UK	1) work, later family	8.9			34.4			
			2) work, cohabitation, later parent	5.1			6.5			
			3) work, marriage, non-parent	8.9			7.8			
			4) work, earlier family	11.7			31.9			
			5) later family, work break	14.0			0.2			
			6) work, no family	10.1			12.8			
			7) earlier family, work break	15.8			0.1			
			8) part-time work, earlier family	18.0			0.3			
			9) no paid work, earlier family	3.3			0.1			
			10) work, divorced parent	2.5			4.2			
			11) teen parent	1.2			0.8			
			12) unstable work, no family	0.6			0.9			

Mooyaart et al. (2019)	1980-1984	US	1) unstable employment-parental home	15.6			29.2			
			2) unstable employment-cohabitation-child	8.2			7.8			
			3) college-employed-married	10.1			7.7			
			4) unstable employment-single-child	22.0			11.4			
			5) unstable employment-married-child	18.1			11.5			
			6) college-employed-cohabitation	10.4			7.4			
			7) college-employed-single	13.1			17.2			
			8) unstable employment-single	3.6			8.7			

Müller et al. (2012)	not reported	Switzerland	1) institutionalized life course							43.0
			2) standardized life course							30.0
			3) unstable life course							27.0

Mynarska et al. (2015)	1965-1974	Italy	1) low-educated single working women	42.3						
			2) married working women	12.5						
			3) disadvantaged women	21.9						
			4) focus on education and work	19.5						
			5) stay-at-home wives	3.8						

		Poland	1) low-educated single working women	17.4						
			2) married working women	17.4						
			3) disadvantaged women	15.8						
			4) focus on work	18.3						
			5) focus on work 2 - continuous education	16.2						
6) women with unstable unemployment	14.9						

Oesterle et al. (2010)	1975	US	**Women**							
			1) unmarried early mothers	27.4						
			2) married mothers	29.3						
			3) postsecondary-educated women without children	43.4						

			**Men**							
			1) unmarried men with limited postsecondary education				26.2			
			2) married fathers				31.6			
3) postsecondary-educated men without children				42.2			

Oesterle et al. (2011)	1975	US	**Women**							
			1) unmarried early mothers	27.4						
			2) married mothers	29.3						
			3) postsecondary-educated women without children	43.4						

			**Men**							
			1) unmarried men with limited postsecondary education				26.2			
			2) married fathers				31.6			
3) postsecondary-educated men without children				42.2			

Pailhé (2013)	1954-1968	France	1) work-oriented, 1 child							9.6
			2) mother works full time, 0 or 1 postponed child							9.6
			3) mother works full time, spaced births							7.5
			4) 2 children and full-time employment							14.1
			5) 3 children and full-time employment							8.1
			6) 2 children and part-time employment							7.1
			7) 3 children and part-time employment							5.7
			8) 1 child and inactivity							6.4
			9) 2 children and inactivity							6.5
			10) 3 children and inactivity							9.4
			11) shift to inactivity, multiple states							15.7

Piccarreta and Billari (2007)*	1960-1968	UK	1) early and high fertility, union, not employed	9.5						
			2) single mothers	5.4						
			3) higher education, combining work and family	12.3						
			4) work after birth of the second child	9.7						
			5) work-oriented, no early union no early single motherhood	7.1						
			6) work-oriented, earlier entry to work	9.0						
			7) early combining work, union, fertility	10.9						
			8) earlier but not higher fertility, no work after the second child	4.7						
			9) late entry labour market, moderately high fertility	1.7						
			10) work and union with limited fertility	11.9						
			11) work and early union with limited fertility	12.1						
			12) low education, early work without family	5.7						

Pollock (2007)	not reported	UK	15, but simplified 10-cluster solution presented							
			1) retired widows who own their house outright							3.3
			2) retired and married own occupiers							16.9
			3) retired local authority renters, many of whom are long term sick or disabled							6.7
			4) workers living in rented accommodation							13.2
			5) young single employees with mortgages							12.8
			6) women workers, responsible for children and mortgages							10.4
			7) older affluent workers who own outright							6.7
			8) self-employed, mostly male and married							5.1
			9) divorced employees with mortgages							4.5
			10) married employees with mortgages							20.4

Sabbath et al. (2015)	1936-1956	US	1) consistently working married mothers	34.5						
			2) married mother who went back to work later	17.4						
			3) married mother who went back to work earlier	15.4						
			4) nonworking married mother	10.4						
			5) working single mother	8.4						
			6) working non mother	7.5						
			7) nonworking single mother	6.5						

Salmela-Aro et al. (2011)	1966-1973	Finland	1) fast starters	14.9			17.1			
			2) fast partnership and late parenthood	16.3			9.8			
			3) career and family	23.4			24.4			
			4) career and unsteady partnership	15.6			14.6			
			5) slow starters	17.0			24.4			
			6) singles with slow careers	12.8			9.8			

Salmela-Aro et al. (2014)	1966-1973	Finland	1) fast starters	14.9			17.1			
			2) fast partnership and late parenthood	16.3			9.8			
			3) career and family	23.4			24.4			
			4) career and unsteady partnership	15.6			14.6			
			5) slow starters	17.0			24.4			
			6) singles with slow careers	12.8			9.8			

Scherger et al. (2016)	1916-1927	UK	1) longer education, later marriage and family formation with continuous career	9.5			34.5			
	1928-1937		2) early marriage and family formation, some short breaks from work	28.4			39.9			
	1938-1947		3) mothers dropping out of the labour market and other non-working married parents	17.2			2.0			
	1948-1957		4) working long-term divorcees with children	7.8			6.0			
			5) childless married (and partly divorced)	7.0			7.8			
			6) mothers with a longer work break, but returning to the labour market	19.7			0.0			
			7) never, briefly or late married and mostly without children	6.4			8.6			
			8) heterogeneous patterns of long-term non-employment	3.9			1.3			

Sirniö et al. (2017)	1972-1975	Finland	1) mid-educated core labour force & typical family formation	32.7			42.1			
			2) mid-educated core labour force & solo or childless couples	17.8			24.6			
			3) express & unsystematic	28.2			6.3			
			4) highly-educated core labour force & late family formation	14.5			11.0			
			5) inactive	5.0			8.8			
			6) late home-leavers	1.8			7.2			

Stafford et al. (2019)	1946	UK	1) work, early family	14.0			49.9			
			2) work, marriage, non-parent	9.1			7.7			
			3) work, no family	5.8			9.8			
			4) work, later family	3.4			30.3			
			5) later family, work break	12.7			1.0			
			6) early family, work break	15.1			0.6			
			7) early family, part-time work	30.7			0.8			
			8) early family, no paid work	9.2			0.01			

Tocchioni (2018)	1907-1969	Italy	**Women**	**1907-44**	**1954-59**	**1960-69**				
			1) disadvantaged	17.5	15.9	18.3				
			2) highly educated, unstable employment and partnership	4.7	9.5	16.1				
			3) employed married	11.6	17.0	9.7				
			4) employed single	28.1	35.6	35.2				
			5) self-employed	14.4	6.8	12.3				
			6) stay at home wives	23.7	15.1	8.4				

			**Men**				**1907-44**	**1954-59**	**1960-69**	
			1) disadvantaged				6.9	9.2	9.7	
			2) highly educated, unstable employment and partnership				11.9	17.4	23.5	
			3) employed married				26.4	19.0	12.9	
			4) employed single				29.0	38.2	36.1	
			5) self-employed married men				12.6	6.1	5.7	
6) self-employed single men				13.1	10.1	12.2	

Van Hedel et al. (2016)	1935-1956	US, SHARE countries		**US**	**Europe**					
			1) working single childless women	10.0	11.2					
			2) nonworking married mothers	24.8	29.3					
			3) working single mothers	11.3	5.5					
			4) married mothers who returned to work after some non-employment	31.2	24.8					
			5) working married mothers	22.7	29.2					

Vidal et al. (2020)	1930-1949	Germany	1) stay-at-home mothers	38.5						
			2) work-focused	3.4						
			3) return to part-time work	7.7						
			4) late work disattachment	27.6						
			5) work and family combined	22.8						

	1958-1981		1) childless	25.5						
			2) late family formation	35.4						
			3) disattachment	7.9						
			4) working mothers (2+ children)	5.0						
			5) extended education	16.3						
6) working mothers (1 child)		10.0						

Worts et al. (2013)	1942-1945	US	1) married mother full timer	27.3						
	1946-1949		2) married mother late entrant	13.0						
	1950-1953		3) married mother gradual entrant	10.0						
	1957-1960		4) married mother part timer	6.6						
	1961-1964		5) empty-nest divorcer	6.5						
			6) married employed non-mother	8.7						
			7) single employed mother	6.8						
			8) single employed non-mother	13.5						
			9) married at-home non-mother	2.2						
			10) single at-home mother	3.6						
			11) single at-home non-mother	1.9						

Xue et al. (2020)	before 1956	UK	1) mixed family, some part-time employment	23.8						
			2) early married parenthood, early domestic labor	14.8						
			3) later married parenthood, later domestic labor	18.2						
			4) later marriage, early full-time employment	13.2						
			5) later marriage/single, later full-time employment	11.4						
			6) single, early full-time employment	18.6						

Zimmermann (2021)	1920-1957	Germany	1) married, children, working part-time continuously	6.0						
			2) married, no children, working full-time	5.0						
			3) single, no children, working full-time	5.0						
			4) married, no children, mostly not working	4.0						
			5) married, child(ren), working full-time	11.0						
			6) married, child(ren), starting part-time work after break, later also full-time work	9.0						
			7) married, child(ren), not working	26.0						
			8) married, child(ren), working part-time after break	13.0						
			9) unmarried, child(ren), discontinuous work	7.0						
			10) married, child(ren), mostly not working	10.0						
			11) not working, child(ren), mostly married, marriage ended around age 50 years	4.0						

* Titles of the trajectories suggested by the authors of this review as the study authors did not ascribed titles to the identified trajectories

## Appendix 1 Search strategy

**EMBASE and MEDLINE**.

(trajector*:ti,ab OR course*:ti OR class*:ti OR group*:ti OR cluster*:ti OR path*:ti OR pattern*:ti OR profile*:ti OR longitudinal:ti OR 'life course'/exp/mj OR 'longitudinal study'/exp/mj) AND (work*:ti OR job*:ti,ab OR occupation*:ti,ab OR profession*:ti OR employ*:ti OR employment:ab OR career:ti,ab OR labour:ti OR labor:ti OR 'work'/exp/mj OR 'occupation'/exp/mj) AND (family:ti,ab OR marri*:ti OR cohabitat*:ti OR union:ti OR parent*:ti OR father*:ti OR mother*:ti OR matern*:ti OR patern*:ti OR child*:ti OR life:ti OR fertil*:ti OR 'homemaking and the family'/exp/mj).

Publication type: article, review, article in press.

**PsycINFO**.

(TI trajector* OR AB trajector* OR TI course* OR TI class* OR TI group* OR TI cluster* OR TI path* OR TI pattern* OR TI profile* OR TI longitudinal) AND (TI work* OR TI job* OR AB job* OR TI occupation* OR AB occupation* OR TI profession* OR TI employ* OR AB employment OR TI career OR AB career OR TI labour OR TI labor OR SU Family Work Relationship) AND (TI family OR AB family OR TI marri* OR TI cohabitat* OR TI union OR TI parent* OR TI father* OR TI mother* OR TI matern* OR TI patern* OR TI child* OR TI life OR TI fertil* OR SU Family Work Relationship).

Publication type: Peer reviewed journal.

Document type: journal article.

**SOCIndex**.

(TI trajector* OR AB trajector* OR TI course* OR TI class* OR TI group* OR TI cluster* OR TI path* OR TI pattern* OR TI profile* OR TI longitudinal) AND (TI work* OR TI job* OR AB job* OR TI occupation* OR AB occupation* OR TI profession* OR TI employ* OR AB employment OR TI career OR AB career OR TI labour OR TI labor OR SU WORK & family) AND (TI family OR AB family OR TI marri* OR TI cohabitat* OR TI union OR TI parent* OR TI father* OR TI mother* OR TI matern* OR TI patern* OR TI child* OR TI life OR TI fertil* OR SU WORK & family).

Document type: article.

Limited to peer reviewed journal.

**Web of Science**.

(TI=trajector* OR TS=trajector* OR TI=course* OR TI=class* OR TI=group* OR TI=cluster* OR TI=path* OR TI=pattern* OR TI=profile* OR TI=longitudinal) AND (TI=work* OR TI=job* OR TS=job* OR TI=occupation* OR TS=occupation* OR TI=profession* OR TI=employ* OR TS=employment OR TI=career OR TS=career OR TI=labour OR TI=labor) AND (TI=family OR TS=family OR TI=marri* OR TI=cohabitat* OR TI=union OR TI=parent* OR TI=father* OR TI=mother* OR TI=matern* OR TI=patern* OR TI=child* OR TI=life OR TI=fertil*).

Type: article, review.

## References

[bib1] Aassve A., Billari F.C., Piccarreta R. (2007). Strings of adulthood: A sequence analysis of Young British Women’s work-family trajectories. European Journal of Population.

[bib2] Aeby G., Gauthier J.-A., Widmer E.D. (2019). Beyond the nuclear family: Personal networks in light of work-family trajectories. Advances in Life Course Research.

[bib3] Ahrens C.J.C., Ryff C.D. (2006). Multiple roles and well-being: Sociodemographic and psychological moderators. Sex Roles.

[bib4] Aisenbrey S., Fasang A. (2017). The interplay of work and family trajectories over the life course: Germany and the United States in comparison. American Journal of Sociology.

[bib5] Amato P.R., Kane J.B. (2011). Life-course pathways and the psychosocial adjustment of young adult women. Journal of Marriage and Family.

[bib6] Amato P.R., Landale N.S., Havasevich-Brooks T.C., Booth A., Eggebeen D.J., Schoen R., McHale S.M. (2008). Precursors of young women’s family formation pathways. Journal of Marriage and Family.

[bib7] Amick B.C., McLeod C.B., Bültmann U. (2016). Labor markets and health: An integrated life course perspective. Scandinavian Journal of Work, Environment & Health.

[bib8] Arpino B., Gumà J., Julià A. (2018). Early-life conditions and health at older ages: The mediating role of educational attainment, family and employment trajectories. PLoS One.

[bib9] Barnett Amanda E. (2013). Pathways of adult children providing care to older parents. Journal of Marriage and Family.

[bib10] Baxter J., Hewitt B., Haynes M. (2008). Life course transitions and housework: Marriage, parenthood, and time on housework. Journal of Marriage and Family.

[bib11] Benach J., Vives A., Tarafa G., Delclos C., Muntaner C. (2016). What should we know about precarious employment and health in 2025? Framing the agenda for the next decade of research. International Journal of Epidemiology.

[bib12] Bennett R., Waterhouse P. (2018). Work and family transitions and the self-rated health of young women in South Africa. Social Science & Medicine.

[bib13] Billari F.C., Liefbroer A.C. (2010). Towards a new pattern of transition to adulthood?. Advances in Life Course Research.

[bib14] Carmichael F., Ercolani M.G. (2016). Unpaid caregiving and paid work over life-courses: Different pathways, diverging outcomes. Social Science & Medicine.

[bib15] Comolli C.L., Bernardi L., Voorpostel M. (2021). Joint family and work trajectories and multidimensional wellbeing. European Journal of Population.

[bib16] Cukrowska-Torzewska E. (2017). Cross-country evidence on motherhood employment and wage gaps: The role of work-family policies and their interaction. Social Politics.

[bib17] Davia Maria A., Legazpe Nuria (2014). Female employment and fertility trajectories in Spain: An Optimal Matching Analysis. Work, Employment and Society.

[bib18] Deole S.S., Zeydanli T. (2021). The causal impact of education on gender role attitudes: Evidence from European datasets. SSRN Electronic Journal.

[bib19] Engels M., Weyers S., Moebus S., Jöckel K.H., Erbel R., Pesch B., Wahrendorf M. (2019). Gendered work-family trajectories and depression at older age. Aging Mental Health.

[bib20] England P. (2010). The gender revolution: Uneven and stalled. Gender and Society.

[bib21] Flores M., Kalwij A. (2014). The associations between early life circumstances and later life health and employment in Europe. Empirical Economics.

[bib22] Fokkema T. (2002). Combining a job and children: Contrasting the health of married and divorced women in the Netherlands?. Social Science & Medicine.

[bib23] Gauthier J.A., Widmer E.D., Bucher P., Notredame C. (2010). Multichannel sequence analysis applied to social science data. Sociological Methodology.

[bib24] Goldscheider F., Bernhardt E., Lappegård T. (2015). The gender revolution: a framework for understanding changing family and demographic behavior. Population and Development Review.

[bib25] Greenhaus J.H., Beutell N.J. (1985). Sources of conflict between work and family roles. Academy of Managment Reviews.

[bib26] Grundy E.M.D., Tomassini C. (2010). Marital history, health and mortality among older men and women in England and Wales. BMC Public Health.

[bib27] Han S.Y., Liefbroer A.C., Elzinga C.H. (2017). Comparing methods of classifying life courses: sequence analysis and latent class analysis. Longitude Life Course Studies.

[bib28] Hayden J.A., Wilson M.N., Riley R.D., Iles R., Pincus T., Ogilvie R. (2019). Individual recovery expectations and prognosis of outcomes in non‐specific low back pain: Prognostic factor review. Cochrane Database of Systematic Reviews.

[bib29] Hegewisch A., Gornick J.C. (2011). The impact of work-family policies on women’s employment: a review of research from OECD countries. Community, Work & Family.

[bib30] Hewitt B., Baxter J., Western M. (2006). The impact of marriage, parenthood and employment on self-reported health of Australian men and women. Journal of Sociology.

[bib31] Huang Q., El-Khouri B.M., Johansson G., Lindroth S., Sverke M. (2007). Women’s career patterns: A study of Swedish women born in the 1950s. Journal of Occupational and Organizational Psychology.

[bib32] Ice E., Ang S., Greenberg K., Burgard S. (2020). Women’s work-family histories and cognitive performance in later life. American Journal of Epidemiology.

[bib33] Janzen B.L., Muhajarine N. (2003). Social role occupancy, gender, income adequacy, life stage and health: A longitudinal study of employed Canadian men and women. Social Science & Medicine.

[bib34] Jin L., Lazar A., Sears J., Todd-Blick A., Sim A., Wu K., Anna Spurlock C. (2020). Clustering life course to understand the heterogeneous effects of life events, gender, and generation on habitual travel modes. IEEE Access.

[bib35] Johansson G., Huang Q., Lindfors P. (2007). A life-span perspective on women’s careers, health, and well-being. Social Science & Medicine.

[bib36] Kalucza S., Hammarström A., Nilsson K. (2015). Mental health and parenthood – A longitudinal study of the relationship between self-reported mental health and parenthood. Health Sociology Review.

[bib37] Kaufman G. (2018). Barriers to equality: why British fathers do not use parental leave. Community, Work & Family.

[bib38] Koelet S., de Valk H., Glorieux I., Laurijssen I., Willaert D. (2015). The timing of family commitments in the early work career: Work-family trajectories of young adults in Flanders. Demographic Research.

[bib39] Kostiainen E., Martelin T., Kestilä L., Martikainen P., Koskinen S. (2009). Employee, Partner, and Mother: Woman’s three roles and their implications for health. Journal of Family Issues.

[bib40] Kuh D., Ben-Shlomo Y., Lynch J., Hallqvist J., Power C. (2003). Life course epidemiology. Journal of Epidemiology and Community Health.

[bib41] Kühhirt M. (2012). Childbirth and the long-term division of labour within couples: how do substitution, bargaining power, and norms affect parents’ time allocation in West Germany?. European Sociological Review.

[bib42] Lacey R.E., Kumari M., Sacker A., Stafford M., Kuh D., McMunn A. (2016). Work-family life courses and metabolic markers in the MRC national survey of health and development. PLoS One.

[bib43] Lacey R.E., Sacker A., Bell S., Kumari M., Worts D., McDonough P., McMunn A. (2017). Work-family life courses and BMI trajectories in three British birth cohorts. International Journal of Obesity.

[bib44] Lacey R.E., Sacker A., Kumari M., Worts D., McDonough P., Booker C., McMunn A. (2016). Work-family life courses and markers of stress and inflammation in mid-life: Evidence from the national child development study. International Journal of Epidemiology.

[bib45] Lacey R.E., Stafford M., Sacker A., McMunn A. (2016). Work-family life courses and subjective wellbeing in the MRC national survey of health and development (the 1946 British birth cohort study). Journal of Population Ageing.

[bib46] Liberati A., Altman D.G., Tetzlaff J., Mulrow C., Gøtzsche P.C., Ioannidis J.P.A., Moher D. (2009). The PRISMA statement for reporting systematic reviews and meta-analyses of studies that evaluate healthcare interventions: explanation and elaboration. BMJ.

[bib47] Macmillan R. (2005). The structure of the life course: Classic issues and current controversies. Advances in Life Course Research.

[bib48] Madero-Cabib I., Fasang A.E. (2016). Gendered work–family life courses and financial well-being in retirement. Advances in Life Course Research.

[bib49] Madero-Cabib Ignacio, Gauthier Jacques Antoine, Le Goff Jean Marie (2016). The influence of interlocked employment-family trajectories on retirement timing. Work, Aging and Retirement.

[bib50] Maier R., Egger A., Barth A., Winker R., Osterode W., Kundi M., Ruediger H. (2006). Effects of short- and long-term unemployment on physical work capacity and on serum cortisol. International Archives of Occupational and Environmental Health.

[bib51] Mayeda E.R., Mobley T.M., Weiss R.E., Murchland A.R., Berkman L.F., Sabbath E.L. (2020). Association of work-family experience with mid- and late-life memory decline in US women. Neurology.

[bib52] McDonough P., Worts D., Booker C., McMunn A., Sacker A. (2015). Cumulative disadvantage, employment-marriage, and health inequalities among American and British mothers. Advances in Life Course Research.

[bib53] McKetta S., Prins S.J., Platt J., Bates L.M., Keyes K. (2018). Social sequencing to determine patterns in health and work-family trajectories for U.S. women, 1968–2013. SSM - Population Health.

[bib54] McMunn A. (2020). Handbook on demographic change and the lifecourse.

[bib55] McMunn A., Lacey R., Worts D., McDonough P., Stafford M., Booker C., Sacker A. (2015). De-standardization and gender convergence in work–family life courses in Great Britain: a multi-channel sequence analysis. Advances in Life Course Research.

[bib56] McMunn A., Lacey R.E., Kumari M., Worts D., McDonough P., Sacker A. (2016). Work-family life courses and metabolic markers in mid-life: Evidence from the British National Child Development Study. Journal of Epidemiology and Community Health.

[bib57] Mills M., Rindfuss R.R., McDonald P., te Velde E. (2011). Why do people postpone parenthood? Reasons and social policy incentives. Human Reproduction Update.

[bib58] Misra J., Budig M., Boeckmann I. (2011). Work-family policies and the effects of children on women’s employment hours and wages. Community, Work & Family.

[bib59] Moen P., Sweet S. (2004). From ‘work–family’ to ‘flexible careers’. Community, Work & Family.

[bib60] Mooyaart J.E., Liefbroer A.C., Billari F.C. (2019). Becoming obese in young adulthood: The role of career-family pathways in the transition to adulthood for men and women. BMC Public Health.

[bib61] Müller N.S., Sapin M., Jacques-Antoine G., Orita A., Widmer E.D. (2012). Pluralized life courses? An exploration of the life trajectories of individuals with psychiatric disorders. International Journal of Social Psychiatry.

[bib62] Mynarska M., Matysiak A., Rybińska A., Tocchioni V., Vignoli D. (2015). Diverse paths into childlessness over the life course. Advances in Life Course Research.

[bib63] OECD (2019). PF2.1 Key characteristics of parental leave systems, OECD - social policy division - directorate of employment. Labour and Social Affairs.

[bib64] Oesterle S., Hawkins J.D., Hill K.G. (2011). Men’s and women’s pathways to adulthood and associated substance misuse. Journal of Studies on Alcohol and Drugs.

[bib65] Oesterle S., Hawkins J.D., Hill K.G., Bailey J.A. (2010). Men’s and women’s pathways to adulthood and their adolescent precursors. Journal of Marriage and Family.

[bib66] Ouzzani M., Hammady H., Fedorowicz Z., Elmagarmid A. (2016). Rayyan—a web and mobile app for systematic reviews. Systematic Reviews.

[bib67] Pailhé A. (2013). Work and family over the life-course. A typology of French long-lasting couples using optimal matching. Longitudinal and Life Course Studies.

[bib68] Piccarreta Raffaella, Billari Francesco C. (2007). Clustering work and family trajectories by using a divisive algorithm. Journal of the Royal Statistical Society. Series A: Statistics in Society.

[bib69] Pollock Gary (2007). Holistic trajectories : A study of combined employment, housing and family careers by using multiple-sequence analysis. Journal of the Royal Statistical Society.

[bib70] Sabbath E., Guevara I.M., Glymour M.M., Berkman L.F. (2015). Use of life course work-family profiles to predict mortality risk among US Women. American Journal of Public Health.

[bib71] Salmela-Aro K., Kiuru N., Nurmi J.E., Eerola M. (2011). Mapping pathways to adulthood among Finnish university students: sequences, patterns, variations in family- and work-related roles. Advances in Life Course Research.

[bib72] Salmela-Aro K., Kiuru N., Nurmi J.E., Eerola M. (2014). Antecedents and consequences of transitional pathways to adulthood among university students: 18-year longitudinal study. Journal of Adult Development.

[bib73] Scherger S., Nazroo J., May V. (2016). Work and family trajectories: changes across cohorts born in the first half of the 20th century. Journal of Population Ageing.

[bib74] Schnittker J. (2007). Working more and feeling better: Women’s health, employment, and family life, 1974-2004. American Sociological Review.

[bib75] Schober P.S. (2013). The parenthood effect on gender inequality: Explaining the change in paid and domestic work when British couples become parents. European Sociological Review.

[bib76] Sieber S.D. (1974). Toward a theory of role accumulation. American Sociological Review.

[bib77] Sigurdardottir H.M., Garðarsdóttir Ó. (2018). Backlash in gender equality? Fathers’ parental leave during a time of economic crisis. Journal of European Social Policy.

[bib78] Simon R.W., Caputo J. (2019). The costs and benefits of parenthood for mental and physical health in the United States: The importance of parenting stage. Society and Mental Health.

[bib79] Sirniö O., Kauppinen T.M., Martikainen P. (2017). Intergenerational determinants of joint labor market and family formation pathways in early adulthood. Advances in Life Course Research.

[bib80] Stafford M., Lacey R., Murray E., Carr E., Fleischmann M., Stansfeld S., McMunn A. (2019). Work-family life course patterns and work participation in later life. European Journal of Ageing.

[bib81] Strandh M., Winefield A., Nilsson K., Hammarström A. (2014). Unemployment and mental health scarring during the life course. European Journal of Public Health.

[bib82] Tocchioni V. (2018). Exploring the childless universe: Profiles of women and men without children in Italy. Demographic Research.

[bib83] van de Schoot R., Sijbrandij M., Winter S.D., Depaoli S., Vermunt J.K. (2017). The GRoLTS-checklist: Guidelines for reporting on latent trajectory studies. Structural Equation Modeling: A Multidisciplinary Journal.

[bib84] Van Hedel K., Mejía-Guevara I., Avendaño M., Sabbath E.L., Berkman L.F., Mackenbach J.P., Van Lenthe F.J. (2016). Work-family trajectories and the higher cardiovascular risk of American women relative to women in 13 European countries. American Journal of Public Health.

[bib85] Vidal S., Lersch P.M., Jacob M., Hank K. (2020). Interdependencies in mothers’ and daughters’ work-family life course trajectories: Similar but different?. Demography.

[bib86] Willitts M., Benzeval M., Stansfeld S. (2004). Partnership history and mental health over time. Journal of Epidemiology and Community Health.

[bib87] Woo K., Zhang Z. (2020). The effect of unemployment in depression by age group: using 12 states’ data from the behavioral risk factor surveillance system. Journal of Korean Academy of Community Health Nursing.

[bib88] Worts D., Sacker A., McMunn A., McDonough P. (2013). Individualization, opportunity and jeopardy in American women’s work and family lives: A multi-state sequence analysis. Advances in Life Course Research.

[bib89] Xue B., Tinkler P., McMunn A. (2020). The long shadow of youth: Girls’ transition from full-time education and later-life subjective well-being in the English longitudinal study of ageing. Journals of Gerontology, Series B.

[bib90] Zagorsky J.L. (2017). Divergent trends in US maternity and paternity leave, 1994–2015. American Journal of Public Health.

[bib91] Zimmermann Okka (2021). A matter of combination? The influence of work-family life courses on life satisfaction at higher ages among German women. Journal of Family Issues.

